# Defining the *Sphagnum* Core Microbiome across the North American Continent Reveals a Central Role for Diazotrophic Methanotrophs in the Nitrogen and Carbon Cycles of Boreal Peatland Ecosystems

**DOI:** 10.1128/mbio.03714-21

**Published:** 2022-02-22

**Authors:** Max Kolton, David J. Weston, Xavier Mayali, Peter K. Weber, Karis J. McFarlane, Jennifer Pett-Ridge, Mark M. Somoza, Jory Lietard, Jennifer B. Glass, Erik A. Lilleskov, A. Jonathan Shaw, Susannah Tringe, Paul J. Hanson, Joel E. Kostka

**Affiliations:** a School of Biological Sciences, Georgia Institute of Technology, Atlanta, Georgia, USA; b School of Earth and Atmospheric Sciences, Georgia Institute of Technology, Atlanta, Georgia, USA; c Biosciences Division, Oak Ridge National Laboratory, Oak Ridge, Tennessee, USA; d Physical and Life Sciences Directorate, Lawrence Livermore National Laboratory, Livermore, California, USA; e Northern Research Station, USDA Forest Service, Houghton, Michigan, USA; f Biology Department, Duke University, Durham, North Carolina, USA; g DOE Joint Genome Institute, Lawrence Berkeley National Laboratory, Berkeley, California, USA; h Environmental Genomics and Systems Biology Division, Lawrence Berkeley National Laboratory, Berkeley, California, USA; i Department of Inorganic Chemistry, University of Vienna, Vienna, Austria; j Leibniz Institute for Food Systems Biology and Chair of Food Chemistry and Molecular and Sensory Science, Technical University of Munich, Freising, Germany; k Chair of Food Chemistry and Molecular and Sensory Science, Technical University of Munich, Freising, Germany; l Center for Microbial Dynamics and Infection, Georgia Institute of Technology, Atlanta, Georgia, USA; University of California, Irvine

**Keywords:** peatland, *Sphagnum* moss, core microbiome, *Methyloferula*, methanotrophy, diazotrophy, rare biosphere, Chip-SIP, keystone species, methane oxidation, microbiome, nitrogen fixation, plant microbiome, stable isotope probing

## Abstract

Peat mosses of the genus *Sphagnum* are ecosystem engineers that frequently predominate over photosynthetic production in boreal peatlands. *Sphagnum* spp. host diverse microbial communities capable of nitrogen fixation (diazotrophy) and methane oxidation (methanotrophy), thereby potentially supporting plant growth under severely nutrient-limited conditions. Moreover, diazotrophic methanotrophs represent a possible “missing link” between the carbon and nitrogen cycles, but the functional contributions of the *Sphagnum*-associated microbiome remain in question. A combination of metagenomics, metatranscriptomics, and dual-isotope incorporation assays was applied to investigate *Sphagnum* microbiome community composition across the North American continent and provide empirical evidence for diazotrophic methanotrophy in *Sphagnum*-dominated ecosystems. Remarkably consistent prokaryotic communities were detected in over 250 *Sphagnum* SSU rRNA libraries from peatlands across the United States (5 states, 17 bog/fen sites, 18 *Sphagnum* species), with 12 genera of the core microbiome comprising 60% of the relative microbial abundance. Additionally, nitrogenase (*nifH*) and SSU rRNA gene amplicon analysis revealed that nitrogen-fixing populations made up nearly 15% of the prokaryotic communities, predominated by *Nostocales* cyanobacteria and *Rhizobiales* methanotrophs. While cyanobacteria comprised the vast majority (>95%) of diazotrophs detected in amplicon and metagenome analyses, obligate methanotrophs of the genus *Methyloferula* (order *Rhizobiales*) accounted for one-quarter of transcribed *nifH* genes. Furthermore, in dual-isotope tracer experiments, members of the *Rhizobiales* showed substantial incorporation of ^13^CH_4_ and ^15^N_2_ isotopes into their rRNA. Our study characterizes the core *Sphagnum* microbiome across large spatial scales and indicates that diazotrophic methanotrophs, here defined as obligate methanotrophs of the rare biosphere (*Methyloferula* spp. of the *Rhizobiales*) that also carry out diazotrophy, play a keystone role in coupling of the carbon and nitrogen cycles in nutrient-poor peatlands.

## INTRODUCTION

Boreal peatlands represent one of the oldest vegetated ecosystems (1). Although peatlands cover approximately 3% of the Earth's land surface area, they store almost one-third of terrestrial soil carbon as recalcitrant peat and play a disproportionately significant role in the atmospheric methane budget (2–4). Extreme environmental conditions in boreal peatlands (low temperatures, flooding, anoxia, nutrient limitation, acidity, and antimicrobial properties of *Sphagnum* biomass) decouple organic matter production and mineralization, resulting in peat accumulation (5). Centuries of stable environmental conditions have resulted in the establishment of ecosystems susceptible to climate change (6, 7). For instance, in peatlands, climate warming effects have been associated with carbon loss and enhanced methane emission (8–13), declines in microbial diversity and activity (10, 14), an increase in fine-root biomass (15), and shifts in vegetation composition (16).

Mosses of the genus *Sphagnum* are among the oldest nonvascular terrestrial plant lineages and have coevolved with their associated microbiota for almost 500 million years (17–19). *Sphagnum* mosses are often abundant in wetlands of the Northern Hemisphere, where they frequently dominate primary productivity (20) and serve as a climate change indicator species (21). Often referred to as “ecosystem engineers,” *Sphagnum* mosses outcompete vascular plants by creating and maintaining acidic conditions (pH 3 to 5) along with efficient nutrient scavenging (22–24). *Sphagnum* mosses lack the root or rhizosphere system present in higher plants. Instead, they interact with the surrounding wetland through an array of dead hyaline cells, constituting up to 90% of the plant's volume (25). Hyaline cells serve as water reservoirs and hubs for rhizosphere-like plant-microbe interactions (18, 26–28), which are essential for plant productivity and ecosystem nutrient cycles (14, 18, 28–37). Therefore, changes in *Sphagnum*-associated microbial communities, which occupy the hyaline cells and leaf surfaces, may constitute early indicators of ecosystem disturbance (14, 38).

The majority of plant microbiome research has centered on the microbiomes of model or crop plants in agricultural systems (39–42), and the microbiomes of wild plants remain less well studied (43, 44). Sequence-based studies have revealed that *Sphagnum*-associated prokaryotic communities are taxonomically diverse (14, 18, 26, 36, 45–48) and differ substantially from surrounding peat soil (9–11, 28, 49). Years of coevolution have yielded particular bacterial assemblages that support plant development, which may be considered a “core microbiome” (18, 49). Core microbiomes contain members with essential genomic traits to support plant growth and ecosystem functioning (50).

Biogeochemical evidence points to an important role for *Sphagnum*-associated prokaryotic populations in mediating critical ecosystem processes such as methane oxidation (methanotrophy) and nitrogen fixation (diazotrophy) (34–36, 48, 51–54). For example, *Sphagnum*-associated methanotrophs act as a natural methane biofilter and provide up to one-third of *Sphagnum* carbon needs (29, 31, 32, 34). Additionally, diazotrophic populations can support plant host growth under nitrogen-limited conditions, supplying up to 35% of the *Sphagnum* nitrogen requirement (26, 33, 34, 52). Further, diazotrophy has been invoked to partially explain the contradictory evidence of high nitrogen content of *Sphagnum* tissues and low environmental nitrogen availability (34–36). However, despite *Sphagnum's* central ecological role in boreal ecosystems, limited efforts have been invested in assessing the functional potential of the *Sphagnum*-associated microbiome (28).

In contrast to biogeochemical investigations, current molecular evidence is contradictory with regard to the predominant *Sphagnum*-associated microbial groups mediating diazotrophy and methanotrophy. Several studies suggested that diazotrophic communities are dominated by *Cyanobacteria* (13, 27, 45, 46), while others pointed to a predominance of *Alphaproteobacteria* (14, 27, 35, 55, 56). Methanotrophic communities are frequently dominated by acidophilic members of the *Beijerinckiaceae* and *Methylocystaceae* families within the *Alphaproteobacteria* (36, 48, 56–59). Many known aerobic methanotrophs shown to be capable of diazotrophy are found within the *Alphaproteobacteria*, including members of the *Beijerinckiaceae* and *Methylocystaceae* detected in the *Sphagnum* microbiome, suggesting that single organisms, diazotrophic methanotrophs, may serve as a functional link between the carbon and nitrogen cycles in *Sphagnum*-dominated peatlands (14, 36, 59). However, the significance of this functional linkage remains unresolved.

Given that methanotrophic and diazotrophic populations may benefit the *Sphagnum* host by providing a substantial fraction of plant tissue carbon and nitrogen (29, 31–34, 52), we hypothesized that these functional guilds represent a key component of the *Sphagnum* core microbiome in nutrient-poor peatlands across North America. We analyzed prokaryotic and diazotrophic communities from 250 individual *Sphagnum* plant gametophyte samples collected from peatlands across the North American continent to test this hypothesis. For a subset of these microbiome samples, dual-isotope tracer (^15^N_2_ + ^13^CH_4_) experiments were combined with metagenomic and metatranscriptomic analyses to characterize active members of the methanotrophic and diazotrophic communities. This integrated analysis revealed that *Sphagnum* microbiomes are remarkably consistent over large spatial scales, with diazotrophy dominated by the cyanobacterial family *Nostocaceaea* (order *Nostocales*) and methanotrophy dominated by the *Beijerinckiaceae* family (order *Rhizobiales*). We conclude that members of the *Rhizobiales* play a central role in the coupling of nitrogen and carbon cycles in *Sphagnum*-dominated peatlands.

## RESULTS

### Taxonomic analysis of *Sphagnum*-associated prokaryotic communities.

**FIG 1:**
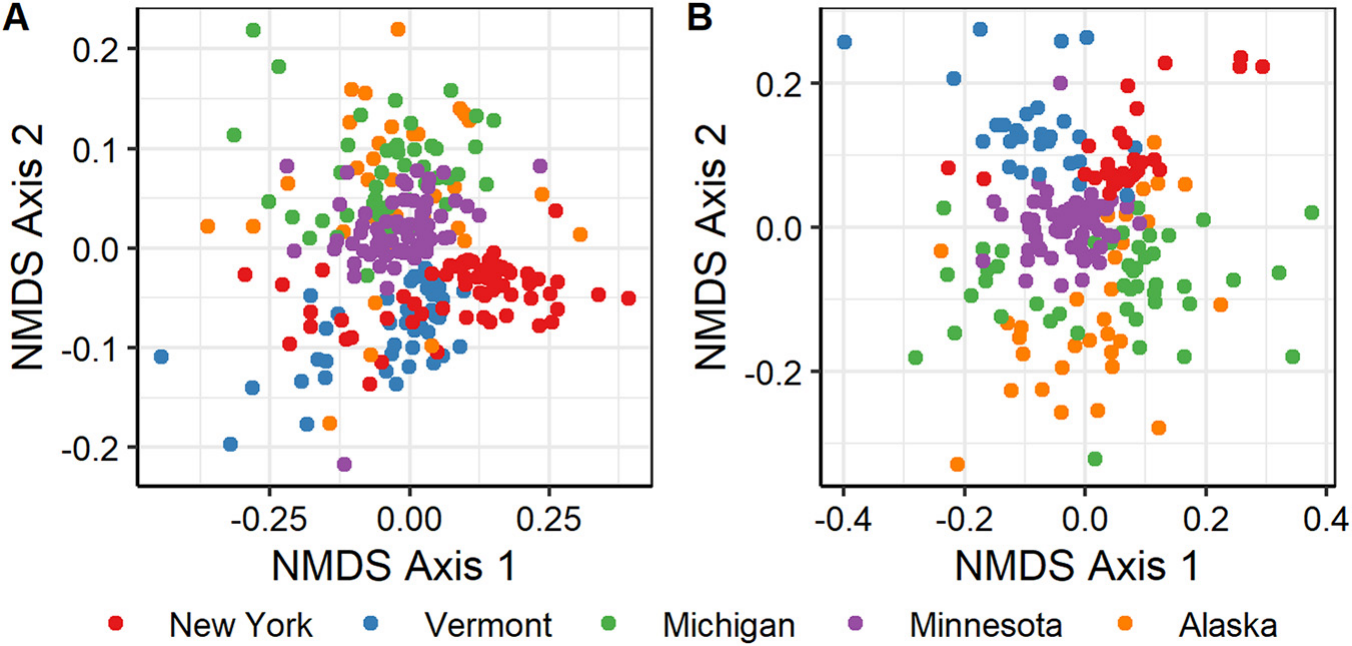
Characterization of the similarity between *Sphagnum*-associated microbial communities across assemblages from different geographical locations. Community similarity is visualized using nonmetric multidimensional scaling (NMDS) of 246 prokaryotic communities based on SSU rRNA gene amplicon sequencing (stress = 0.208) (A) and 195 diazotrophic communities based on *nifH* gene amplicon sequencing (stress = 0.248) (B). High-quality sequence data sets were normalized by cumulative sum scaling (CSS), and beta diversity indices were estimated based on weighted UniFrac distances. A PERMANOVA test on weighted UniFrac distance metrics with 1,000 permutations analyzed significant differences in beta diversity. Different colors represent microbial communities collected from different geographical locations.

We investigated bacterial/archaeal (via small subunit [SSU] 16S rRNA gene sequencing) and diazotrophic (via *nifH* gene sequencing) diversity and taxonomic composition across five U.S. states and 17 bog/fen sites, covering 18 *Sphagnum* species (see Table S1 at https://zenodo.org/record/5786378). We observed high similarity in the *Sphagnum*-associated bacterial/archaeal and diazotrophic communities across the North American continent (Fig. 1; see also Fig. S1 and S2 in the supplemental material and Tables S4 and S5 at https://zenodo.org/record/5786378). Nonmetric multidimensional scaling (NMDS) and permutational multivariate analysis of variance (PERMANOVA) analyses highlight the substantial effects of geographical location and plant species on microbial community structure. Geographical location explained approximately 10% (*R*^2^ = 0.1, *P* < 0.001) and 28% (*R*^2^ = 0.28, *P* < 0.001) of the variation in the bacterial/archaeal and diazotrophic community composition, respectively (Fig. 1). *Sphagnum* species explained ∼10% of the variation in the prokaryotic community (*R*^2^ = 0.1, *P* < 0.001) (Fig. 1A; Fig. S1A; Table S3 at https://zenodo.org/record/5786378) but did not affect diazotrophic community composition (*R*^2^ = 0.08, *P* = 0.07) (Fig. 1B; Fig. S1B; Table S3). The interaction effect between geographical location and *Sphagnum* species was able to explain only an additional 3% of the variation in prokaryotic communities (*R*^2^ = 0.03, *P* = 0.04) (Table S3) and failed to resolve any variation in diazotrophic communities (*R*^2^ = 0.03, *P* = 0.22) (Fig. 1; Table S3). The SSU rRNA-based analysis indicated that prokaryotic communities were dominated by *Proteobacteria* (62% ± 3%), *Acidobacteriota* (14% ± 2.5%), *Cyanobacteria* (8% ± 5%), WPS-2 (4% ± 1%), and *Verrucomicrobiota* (3 ± 0.3%) phyla (Fig. S1; Tables S4 and S5 at https://zenodo.org/record/5786378).

10.1128/mbio.03714-21.2 FIG S1 Characterization of the similarity between *Sphagnum*-associated microbial communities across assemblages from different *Sphagnum* species. Community similarity is visualized using nonmetric multidimensional scaling (NMDS) of 246 prokaryotic communities based on SSU rRNA gene amplicon sequencing (stress = 0.208) (A) and 195 diazotrophic communities based on *nifH* gene amplicon sequencing (stress = 0.248) (B). High-quality sequence data sets were normalized by cumulative sum scaling (CSS), and beta diversity indices were estimated based on weighted UniFrac distances. A PERMANOVA test on weighted UniFrac distance metrics with 1,000 permutations analyzed significant differences in beta diversity. Different colors represent microbial communities collected from different *Sphagnum* species. Download FIG S1, TIF file, 0.3 MB. Copyright © 2022 Kolton et al. 2022 Kolton et al. https://creativecommons.org/licenses/by/4.0/ This content is distributed under the terms of the Creative Commons Attribution 4.0 International license.

10.1128/mbio.03714-21.3 FIG S2 Relative abundance of sequences affiliated with major phyla and orders in *Sphagnum*-associated microbial communities. Samples were grouped according to their characteristics: geographical location (A and D), collection site (B and E), or *Sphagnum* genotype (C and F). The top row represents bacterial phyla, and the bottom row represents bacterial orders. The *Sphagnum*-associated microbial communities are dominated by *Proteobacteria*, followed by *Acidobacteriota* and *Cyanobacteria*. Relative abundances were calculated from 246 SSU rRNA gene amplicon profiles of prokaryotic communities. Download FIG S2, TIF file, 1.0 MB. Copyright © 2022 Kolton et al. 2022 Kolton et al. https://creativecommons.org/licenses/by/4.0/ This content is distributed under the terms of the Creative Commons Attribution 4.0 International license.

**FIG 2:**
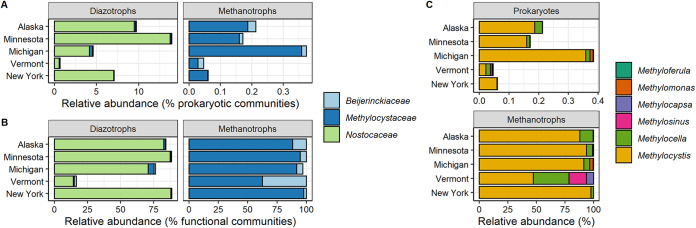
Relative abundance of putative diazotrophic and methanotrophic families in *Sphagnum*-associated microbial communities calculated from prokaryotic (A) or functional guild (B) communities; (C) relative abundance of methanotrophic genera partitioned from prokaryotic (upper panel) or functional guild (lower panel) communities. The functional guild relative abundances were calculated based on the SSU rRNA gene amplicons taxonomically affiliated with putative diazotrophs and/or methanotrophs at the genus level. The *Sphagnum*-associated diazotrophic and methanotrophic communities are dominated by *Nostocaceaea* and *Methylocystaceae* families, respectively. Relative abundances were calculated from 246 SSU rRNA gene amplicon profiles of the prokaryotic communities.

Due to the vital role of the diazotrophic and methanotrophic communities in *Sphagnum* primary productivity, we focused our subsequent analyses on prokaryotic taxa with known diazotrophic and methanotrophic capabilities. Approximately 9.2% and 0.3% of the *Sphagnum*-associated microbial community were affiliated with known putative diazotrophic and/or methanotrophic species, respectively (Fig. S3A and B; Table S6 at https://zenodo.org/record/5786378). The SSU rRNA and *nifH*-based community composition analyses suggested that the cyanobacterial family *Nostocaceaea* (order *Nostocales*) dominates the diazotrophic community (Fig. 2A and B; Fig. S4B; Table S6). The SSU rRNA analyses show that members of the *Methylocystaceae* and *Beijerinckiaceae* families (order *Rhizobiales*) were dominant among methanotrophic populations (Fig. 2C; Table S6). However, the taxonomic composition of diazotrophic and methanotrophic communities varies substantially between geographical locations (Fig. 2; Table S6). For example, diazotrophic members of the *Nostocaceae* family comprised 13.7% ± 1.3% of the total prokaryotic community in Minnesota but contributed only 0.6% ± 0.2% of the *Sphagnum*-associated communities from the Vermont area (Table S6). Similarly, members of methanotrophic communities showed 10-fold variation in their relative abundances. While the relative abundance of the *Methylocystaceae* family was 0.36% ± 0.07% in the Michigan area, their relative abundance in the Vermont area was 0.03% ± 0.01% only (Fig. 2; Table S6).

10.1128/mbio.03714-21.4 FIG S3 Relative abundance of putative diazotrophic and methanotrophic communities. The functional guilds’ relative abundances were calculated based on SSU rRNA gene amplicons (A) or metagenomic contigs taxonomically affiliated with putative diazotrophs and/or methanotrophs at the genus level (B). Approximately 15% and 0.6% of the *Sphagnum*-associated microbial community represent members of the putative diazotrophic and/or methanotrophic communities, respectively. Relative abundances were calculated from 246 SSU rRNA gene amplicon profiles of the prokaryotic communities and 12 metagenome/metatranscriptome libraries. MetaT, metatranscriptome; MetaG, metagenome. Download FIG S3, TIF file, 0.3 MB. Copyright © 2022 Kolton et al. 2022 Kolton et al. https://creativecommons.org/licenses/by/4.0/ This content is distributed under the terms of the Creative Commons Attribution 4.0 International license.

10.1128/mbio.03714-21.5 FIG S4 Relative abundance of putative diazotrophic communities. The composition of diazotrophic communities was inferred from 195 *nifH* gene amplicon libraries. (A) Distribution of *nifH* clusters; (B) taxonomic composition for the functional *nifH* genes (clusters I to III) at the family level; (C) taxonomic distribution of the *Nostocales* at the genus level. Members of the *Nostocaceae* family are dominant members of the *Sphagnum*-associated diazotrophic community. Download FIG S4, TIF file, 0.3 MB. Copyright © 2022 Kolton et al. 2022 Kolton et al. https://creativecommons.org/licenses/by/4.0/ This content is distributed under the terms of the Creative Commons Attribution 4.0 International license.

### The *Sphagnum* core microbiome.

**FIG 3:**
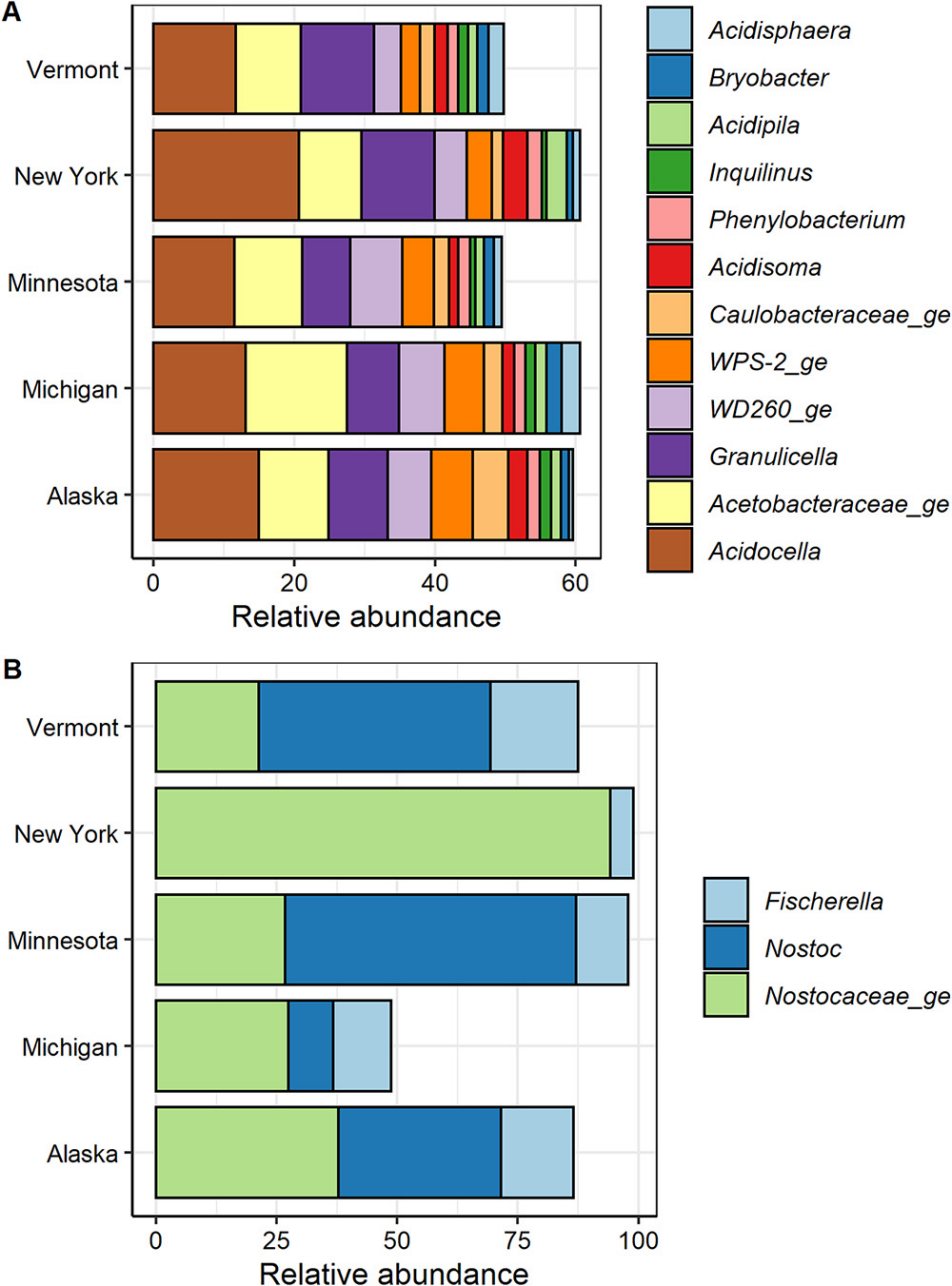
Relative abundance and taxonomic composition of the core microbiomes. (A and B). Relative abundance of the core microbiome at the genus level of prokaryotic (A) and diazotrophic (B) communities. Prokaryotic core microbiome was calculated based on genera shared between 100% of the samples. Diazotrophic core microbiome was calculated based on genera shared between 50% of the samples. Relative abundances were calculated from 246 SSU rRNA and 195 *nifH* gene amplicon profiles of the prokaryotic and diazotrophic communities, respectively. WD260_ge and WPS-2_ge represent candidate genera of the corresponding phyla.

The core microbiome, defined as the collection of community members observed in all *Sphagnum* samples, contained only 7 out of 12,044 amplicon sequence variants (ASVs) (0.06% of the total ASVs) but comprised 12.1% of the relative abundance of the total rRNA gene amplicon sequences retrieved (Fig. S5A and S6A; Table S7 at https://zenodo.org/record/5786378). Similarly, core microbiome analysis at the genus level indicates that 12 bacterial genera contributed nearly 60% of the total sequences (Fig. 3A; Fig. S6B). The *Sphagnum* core microbiome was dominated by *Acidocella*, *Granulicella*, and WPS-2, followed by *Acidisoma*, *Bryobacter*, *Acidisphaera*, and *Phenylobacterium* genera (Fig. 3A; Table S7A). Although the core microbiome at the genus level did not include known methanotrophic genera, analyses at the family level show that the methanotroph-containing *Beijerinckiaceae* and *Methylacidiphilaceae* contribute 1.7% ± 0.2% and 1.6% ± 0.5% of the *Sphagnum*-associated microbiome, respectively (Table S7B). In contrast, the diazotrophic community was less conserved. After omission of the nonfunctional *nifH* cluster IV-V from further analysis, only three ASVs affiliated with *Nostocales* were common across 50% of the samples (Fig. S4C and S5B). Nevertheless, a core microbiome analysis at the genus level revealed that *Nostoc* and *Fischerella* comprised approximately 85% of the total diazotrophic communities (Fig. 3B).

10.1128/mbio.03714-21.6 FIG S5 (A) Relative abundance of bacterial species observed in 100% of the sampling effort. Collectively, seven universally distributed ASVs contribute approximately 12% of the *Sphagnum*-associated microbial community. Relative abundances were calculated from 246 SSU rRNA gene amplicon profiles of the prokaryotic communities. (B) Relative abundance of diazotrophic species observed in 50% of the sampling effort. The taxonomic affiliation of each species at the order and genus levels is indicated on the panel strips. WD260 and WPS-2 represent candidate phyla. Relative abundances were calculated from 195 *nifH* gene amplicon profiles of the diazotrophic communities. Download FIG S5, TIF file, 0.7 MB. Copyright © 2022 Kolton et al. 2022 Kolton et al. https://creativecommons.org/licenses/by/4.0/ This content is distributed under the terms of the Creative Commons Attribution 4.0 International license.

10.1128/mbio.03714-21.7 FIG S6 Accumulated relative abundance of the bacterial core microbiome in different geographical locations. Box plot illustrating the cumulative relative abundance of the microbial core microbiome at the ASV (A and C) and genus (B and D) levels in different geographical locations. Cumulative abundances were determined by taxon prevalence cutoff thresholds at 10% intervals from 50% to 100% for prokaryotic communities (A and B) and from 10% to 55% for diazotrophic communities (C and D). Relative abundances were calculated from 246 SSU rRNA and 195 *nifH* gene amplicon profiles of the prokaryotic and diazotrophic communities, respectively. Download FIG S6, TIF file, 0.7 MB. Copyright © 2022 Kolton et al. 2022 Kolton et al. https://creativecommons.org/licenses/by/4.0/ This content is distributed under the terms of the Creative Commons Attribution 4.0 International license.

### Metagenomic and metatranscriptomic analyses of the *Sphagnum* microbiome.

Triplicate individual plants of Sphagnum fallax and Sphagnum magellanicum were collected in August 2015 from the SPRUCE experimental site at the S1 bog in the Marcell Experimental Forest (http://mnspruce.ornl.gov). Metagenomic/transcriptomic libraries were prepared, sequenced, and analyzed (Text S1; Fig. S7; Table S8 at https://zenodo.org/record/5786378). High-quality reads were coassembled into 3.4 million contigs with a total length of 1.6 Gbp, encoding approximately 3.8 million predicted proteins (Fig. S7). The resulting assembly recruited about 40% and 80% of the metagenomic and metatranscriptomic high-quality reads, respectively.

10.1128/mbio.03714-21.1 TEXT S1 (1) Sample preparation, DNA extraction, PCR, and sequencing. (2) Amplicon data processing. (3) Metagenomic and metatranscriptomic nucleic acid extraction and sequencing. (4) Metagenomic and metatranscriptomic quality control. (5) Illumina data assembly and annotation. (6) Chip-stable isotope probing (Chip-SIP), linking phylogeny with function. Download Text S1, DOCX file, 0.1 MB. Copyright © 2022 Kolton et al. 2022 Kolton et al. https://creativecommons.org/licenses/by/4.0/ This content is distributed under the terms of the Creative Commons Attribution 4.0 International license.

10.1128/mbio.03714-21.8 FIG S7 Summary of metagenomic and metatranscriptomic assembly. (A and B) Relative abundance of reads retained in the sample after the quality control steps for metagenomics (A) and metatranscriptomics (B); (C) total number of contigs discovered after coassembly; (D) total contig length; (E) number of predicted ORFs assigned to protein databases; (F) summary of assembled contigs and predicted proteins. Coassembly was performed with 12 metagenome/metatranscriptome libraries. S.mag, *S. magellanicum*; S.fal, *S. fallax*; MT, metatranscriptome; MG, metagenome. Download FIG S7, TIF file, 0.4 MB. Copyright © 2022 Kolton et al. 2022 Kolton et al. https://creativecommons.org/licenses/by/4.0/ This content is distributed under the terms of the Creative Commons Attribution 4.0 International license.

**FIG 4:**
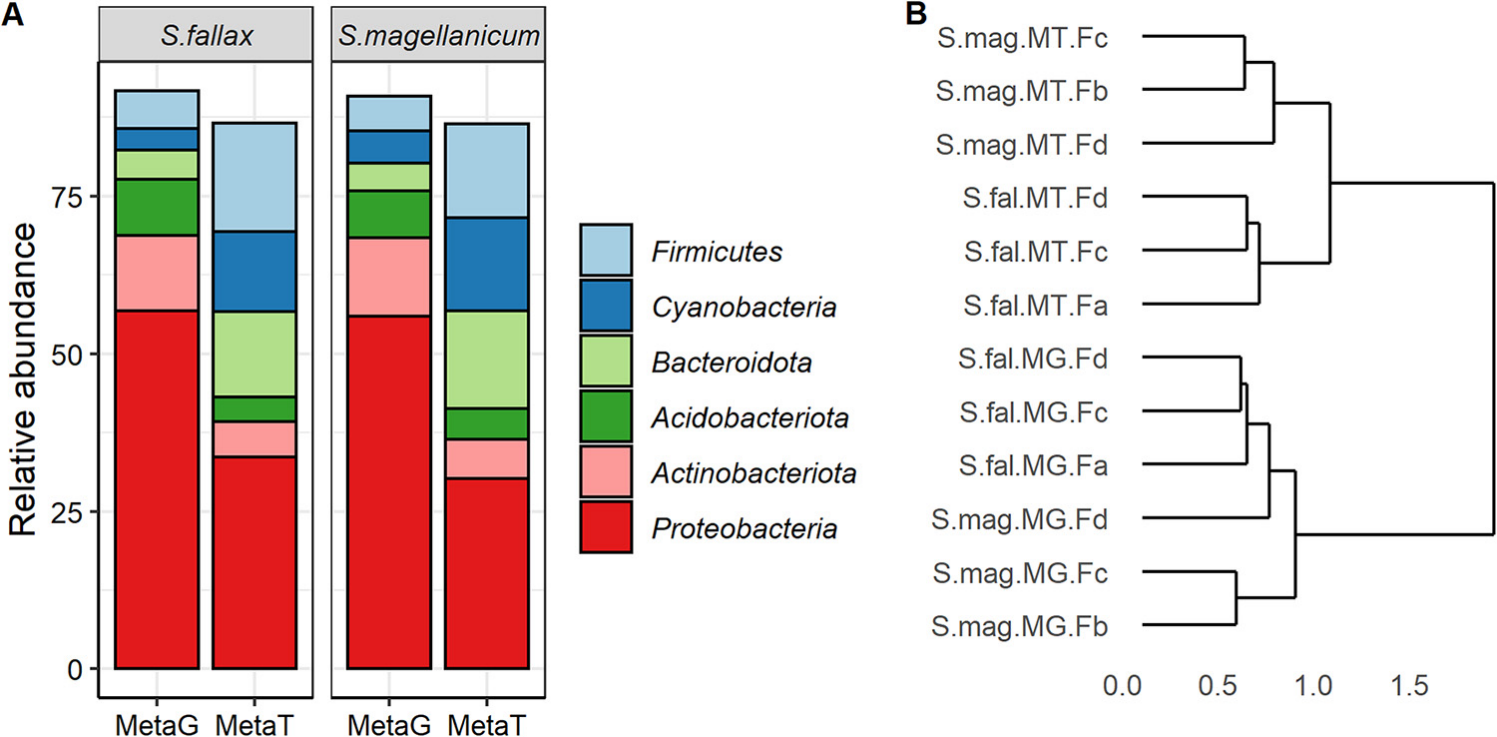
(A) Relative abundance of dominant bacterial phyla in metagenomes and metatranscriptomes of the *Sphagnum*-associated microbial communities. (B) Hierarchical cluster analysis of the *Sphagnum*-associated prokaryotic communities. Hierarchical clustering was performed using a complete linkage on Bray-Curtis distance measures of 1,206,693 prokaryotic contigs. Abbreviations: S.mag, *S. magellanicum*; S.fal, *S. fallax*; MetaT, metatranscriptome; MetaG, metagenome. The indexes Fa, Fb, Fc, and Fd indicate independent plant replicates used for nucleic acid extraction and meta-omics analyses.

The taxonomic composition of the metagenomic libraries correlated well with taxonomy inferred from the SSU rRNA gene analysis, showing the dominance of *Proteobacteria* (56.4%) and *Acidobacteria* (8.2%) (Fig. 4A; Table S9 at https://zenodo.org/record/5786378). However, the taxonomic composition of metagenomic and metatranscriptomic communities differed substantially (Fig. 4A; Fig. S8; Table S9). For example, *Proteobacteria* (31.9%) and *Acidobacteria* (4.4%) phyla were less active than expected based on the metagenomic analysis (56.4% and 8.2%, respectively). In contrast, members of the *Cyanobacteria* (3.7%) and *Bacteroidota* (14.5%) phyla were more abundant in the metatranscriptome libraries than in the metagenomic samples, with 4.3% and 4.5% for *Cyanobacteria* and *Bacteroidota*, respectively (Fig. 4A; Fig. S9; Table S9). Hierarchical cluster analysis of the metagenome and metatranscriptome samples revealed the relationships between the genes and their transcripts in the *Sphagnum*-associated prokaryotic communities (Fig. 4B; Fig. S9). The bacterial/archaeal communities were segregated into two major clusters. The first cluster included all metagenomic samples and was well separated from the metatranscriptomic cluster. Additionally, samples in the metatranscriptomic cluster were grouped into two host-specific subclusters (Fig. 4B). The separation of these samples was confirmed by an independent cluster analysis of the total prokaryotic reads and encoded protein sequences (Fig. S9).

10.1128/mbio.03714-21.9 FIG S8 Relative abundance of dominant bacterial phyla in metagenomes and metatranscriptomes of the *Sphagnum*-associated microbial communities. Pairwise comparisons were completed using Wilcoxon tests. Significance: *, *P* < 0.05; **, *P* < 0.01; ***, *P* < 0.001; ns, not significant. MetaT, metatranscriptome; MetaG, metagenome. Download FIG S8, TIF file, 0.4 MB. Copyright © 2022 Kolton et al. 2022 Kolton et al. https://creativecommons.org/licenses/by/4.0/ This content is distributed under the terms of the Creative Commons Attribution 4.0 International license.

10.1128/mbio.03714-21.10 FIG S9 Hierarchical cluster analysis of the *Sphagnum*-associated prokaryotic communities. Hierarchical clustering was performed using complete linkage on Bray-Curtis distance of 15,526,855 reads (A), 1,206,693 contigs (B), or 1,037,594 ORFs (C). The indexes Fa, Fb, Fc, and Fd indicate independent plant replicates used for nucleic acid extraction and meta-omics analyses. S.mag, *S. magellanicum*; S.fal, *S. fallax*; MT, metatranscriptome; MG, metagenome. Download FIG S9, TIF file, 0.3 MB. Copyright © 2022 Kolton et al. 2022 Kolton et al. https://creativecommons.org/licenses/by/4.0/ This content is distributed under the terms of the Creative Commons Attribution 4.0 International license.

**FIG 5:**
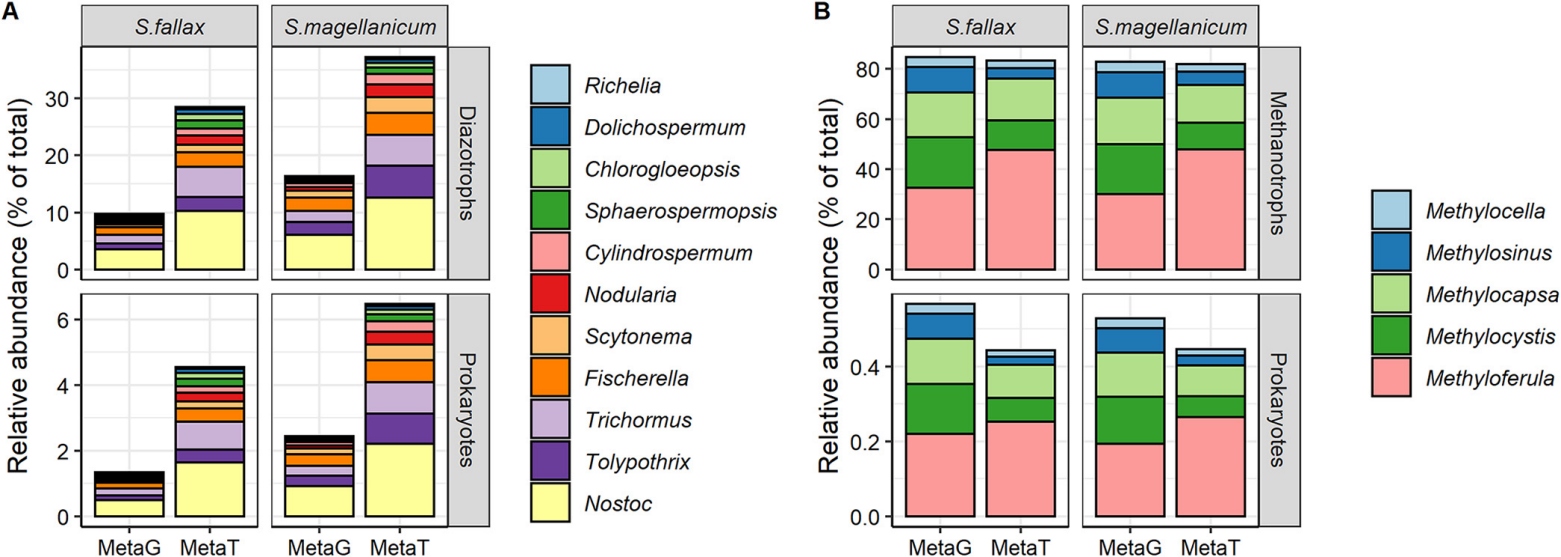
The relative abundances of putative diazotrophic genera within *Nostocaceaea* (A) and methanotrophic genera in *Sphagnum*-associated microbial communities (B) were determined from 6 metagenomes and 6 metatranscriptomes. The functional guild relative abundances were calculated by mapping high-quality reads onto contigs taxonomically affiliated with putative diazotrophs and/or methanotrophs at the genus level, and RPKM (reads per kilobase million) counts were calculated to estimate the abundances of each contig in samples. The upper panel of each plot represents the taxon abundances relative to that of putative functional guilds. The bottom panel represents taxon abundances relative to that of total prokaryotic communities. MetaT, metatranscriptome; MetaG, metagenome.

In remarkable agreement with the SSU and *nifH* gene taxonomic analyses discussed above, the metagenomic analysis indicates that putative diazotrophic and methanotrophic populations contributed approximately 15% ± 2% and 0.6% ± 0.1% of the *Sphagnum*-associated prokaryotic communities, respectively (Fig. S3B). Moreover, the cyanobacterial family *Nostocaceae* dominated the diazotrophic community (Fig. 5A). While the relative abundance of *Nostocaceae*-affiliated contigs comprised 13% ± 3% of the putative diazotrophs, almost 33% ± 3% of the transcriptionally active community was taxonomically affiliated with *Nostocaceae* (Fig. 5A). Additionally, a taxonomic analysis of the methanotrophic community highlighted a central role for the nitrogen-fixing and methane-oxidizing *Rhizobiales*. Approximately 84% ± 2% (Fig. 5B) of the methanotrophic community members are taxonomically affiliated with this order.

Methanotrophic members of the *Beijerinckiaceae* and *Methylocystaceae* families contributed 54% ± 4% and 30% ± 2% of the identified methanotrophic populations based on metagenomes, respectively. However, these two families represented 67% ± 6% and 16% ± 2% of the active methanotrophic communities, respectively. While most of the active methanotrophic populations had similar or lower than predicted abundances based on the metagenomic analysis, one exception was the obligate methanotroph from the genus *Methyloferula* (order *Rhizobiales*), which was significantly more transcriptionally active than expected (31% ± 4% versus 48% ± 6%) (Fig. 5B).

**FIG 6:**
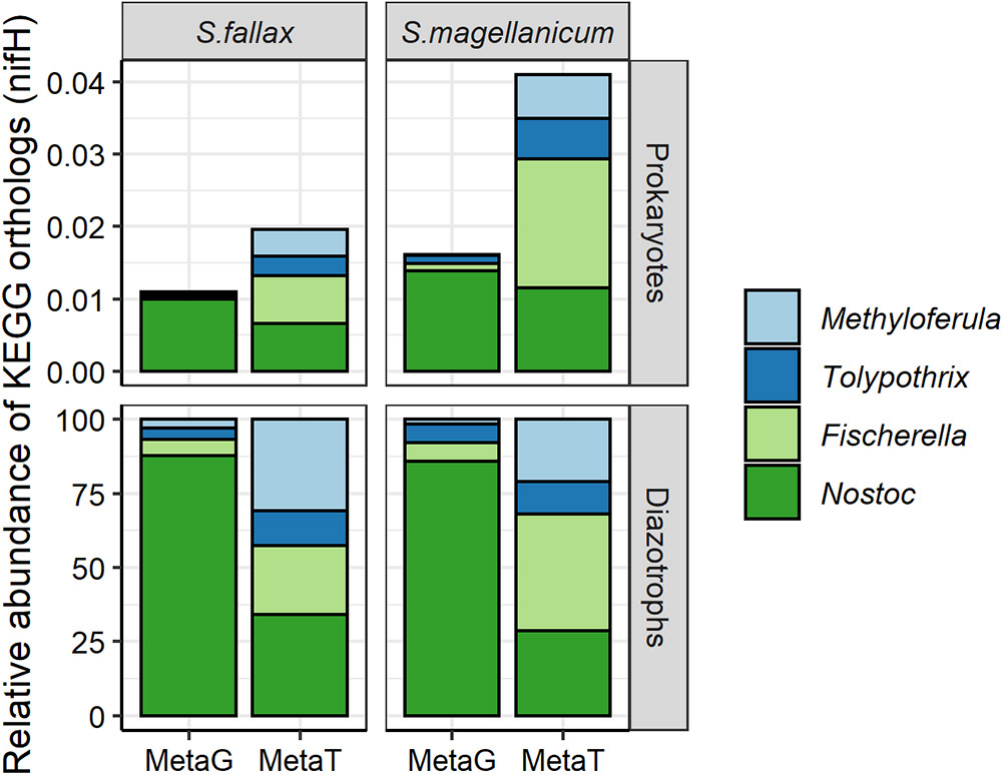
The relative abundances of *nifH* genes at the genus level were determined from 6 metagenomes and 6 metatranscriptomes. High-quality reads were mapped onto *nifH* ORFs, and RPKM (reads per kilobase million) counts were calculated to estimate the abundances of each of the *nifH* ORFs in samples. The bottom panel of each plot represents the taxon abundances relative to that of putative functional guilds. The upper panel shows taxon abundances relative to that of total prokaryotic communities. MetaT, metatranscriptome; MetaG, metagenome.

The *nifH*-encoded protein represented a small portion of the detected open reading frames. Collectively, molybdenum-, vanadium-, and iron-dependent nitrogenase isoforms represented only 0.01% ± 0.01% and 0.03% ± 0.02% of the total KEGG-identified proteins in the metagenomic and metatranscriptomic libraries, respectively (Fig. 6). Furthermore, although at the DNA level almost 98% of the *nifH* genes were taxonomically affiliated with the cyanobacterial genus *Nostoc*, their contribution to the expressed *nifH* genes pool was only 32% ± 11% (Fig. 6). In contrast, the relative abundance of the *nifH* gene from obligate methanotrophs of the genus *Methyloferula* was only 2.2% ± 3.2% in the metagenomic libraries but represented 26% ± 11% of the total transcribed *nifH* genes (Fig. 6).

### Linking phylogeny with function by Chip-SIP analysis.

**FIG 7:**
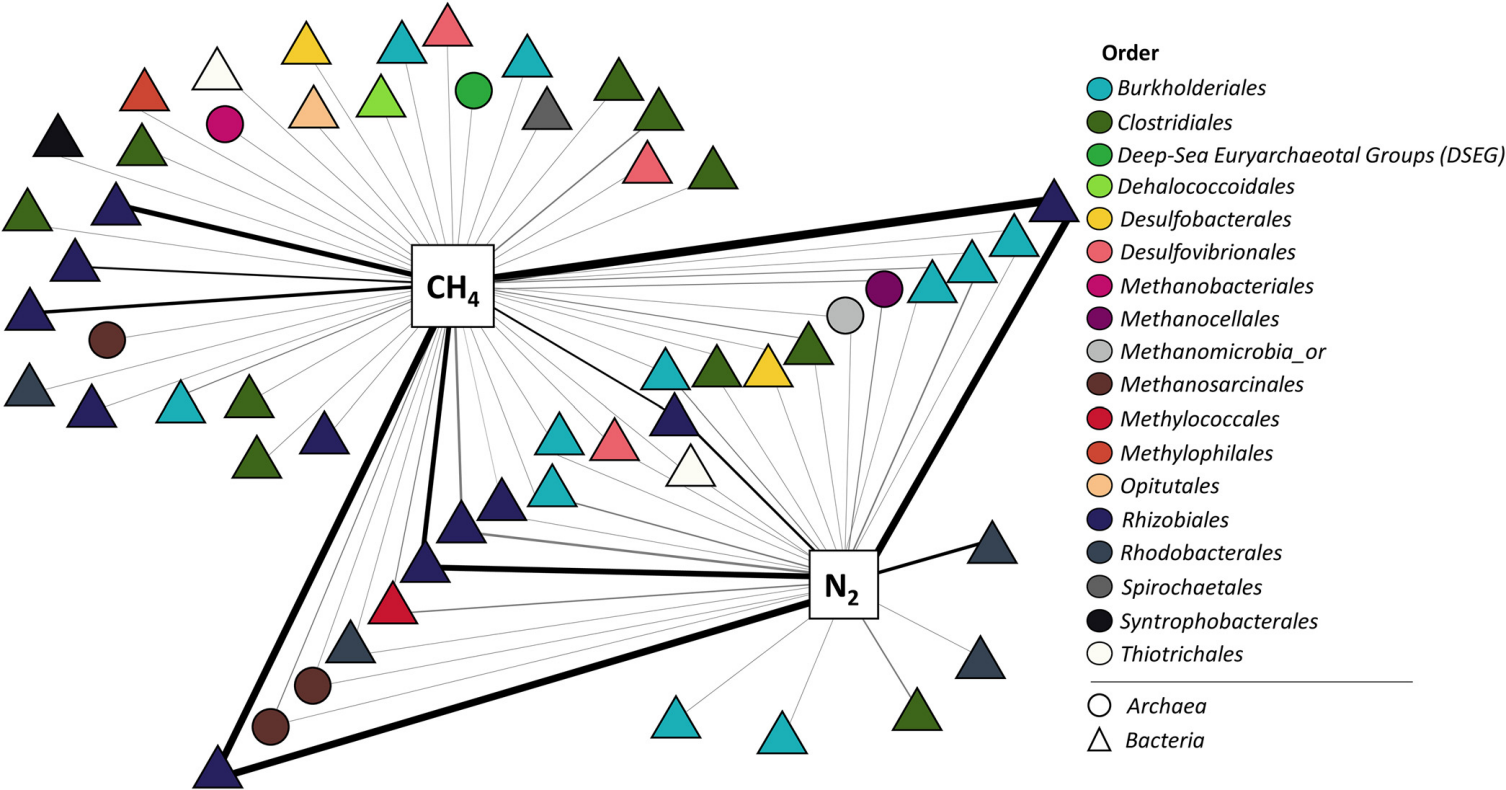
Bipartite network diagram connecting microbial species and isotopically labeled substrates incorporated, as detected by Chip‐SIP analysis of 7 incubations. Triangles and circles represent individual bacterial and archaeal species, respectively, that showed significant incorporation of one or more isotopes. Microbial species are colored according to their taxonomic order assignment. The thickness of the lines is proportional to isotopic incorporation (hybridization‐corrected enrichment [HCE]), normalized to the highest value for each sample and averaged across samples where isotope incorporation was detected. Members of the *Beijerinckiaceae*, *Rhizobiaceae*, and *Bradyrhizobiaceae* families were among the most active members of the diazotrophic community able to oxidize methane simultaneously.

A total of 10 *Sphagnum* samples were incubated with ^13^CH_4_ and ^15^N_2_ for 12 days. Incorporation of the ^15^N and ^13^C isotopes into SSU rRNA transcripts was quantified using Chip-SIP, a type of phylogenetic microarray isotope enrichment analysis (60–62), as a measure of diazotrophic and methanotrophic activities, respectively. The Chip-SIP analysis targeted taxa with the potential for either or both processes. We detected positive isotope incorporation in 7 of 10 samples. Approximately 14% of the taxa (56 of 392 taxa targeted by the array) were enriched above background levels with at least one stable isotope in at least one of these seven samples. Of these 56 labeled taxa, 28 and 5 taxa incorporated ^13^C and ^15^N isotopes into transcribed SSU rRNA, respectively. The remaining 23 taxa (41%) incorporated both isotopes in at least one sample (Table S10 at https://zenodo.org/record/5786378). In addition, a bipartite network analysis connecting microbial species and isotopically labeled substrates indicated that the taxa that were most reliably isotope enriched with ^15^N and ^13^C were in the family *Bradyrhizobiaceae* (*Rhizobiales*) (Fig. 7). Other taxa frequently identified as diazotrophs were in the *Methylocystaceae* (*Rhizobiales*) and *Alcaligenaceae* (*Burkholderiales*), and those frequently identified as methanotrophs were in the *Methanosarcinaceae* (*Archaea*) and *Alcaligenaceae* (*Burkholderiales*). The highest levels of ^15^N or ^13^C enrichment (or both) were measured in *Rhizobiales*. Of 7 taxa with such requirements, four were from the *Rhizobiales* (Fig. 7; Table S10). Members of the *Beijerinckiaceae* family were among the taxa with the highest level of ^13^C incorporation (Fig. 7). Moreover, other representatives of the *Rhizobiales* order, primarily members of the *Beijerinckiaceae*, *Rhizobiaceae*, and *Bradyrhizobiaceae* families, were among the most active members of the diazotrophic community with the ability to simultaneously oxidize methane, as evidenced by dual isotopic ^15^N and ^13^C labeling (Fig. 7).

## DISCUSSION

*Sphagnum* mosses thrive in peatlands despite severe nutrient limitation. A growing body of evidence shows that *Sphagnum* mosses house a diverse microbiome community with the potential to alleviate nitrogen limitation through diazotrophy (18, 28). Furthermore, *Sphagnum*-associated diazotrophs might represent a “missing link” between peatland carbon and nitrogen cycles (18, 63), whereby diazotrophs capable of methanotrophy act as a biofilter, consuming methane and preventing its release to the atmosphere (63). Therefore, diazotrophic methanotrophs have the potential to closely couple the carbon and nitrogen cycles of peatlands.

While the role of *Sphagnum* as an ecosystem engineer in peatlands has been established (16), contributions of the *Sphagnum*-associated microbiomes to ecosystem nutrient cycling processes are still yet to be fully determined. Moreover, studies of the functional potential of *Sphagnum* microbiomes have been limited to relatively few sites and *Sphagnum* species. Here we improve understanding by defining the *Sphagnum* core microbiome over large scales across the North American continent. Additionally, our study reveals that obligate methanotrophs capable of diazotrophy have high nitrogen fixation gene activity levels and incorporate a substantial amount of methane carbon and nitrogen from N_2_ into their biomass.

### The core microbiome of *Sphagnum* spp.

This study reveals remarkably conserved microbial communities associated with a broad range of *Sphagnum* species across U.S. peatlands. In general, our results agree with previous studies of the taxonomic diversity of *Sphagnum* microbiomes (14, 26–28, 48). Host specificity and environmental parameters, which tend to be site specific, are selective forces shown to drive plant microbiome community composition in peatlands (49, 64). The geographic scale and scope (number of *Sphagnum* species, habitat) of microbial data sets may impact the ability to detect community composition variation. Microbiome investigations in a few representative peat moss species in Austrian and Dutch bogs showed that microbiome diversity was both site and host species specific, and host specificity was independent of geographic location (27, 46, 65). Further, in a study of multiple moss species (including *Sphagnum*) across Alaska, microbiome community composition was strongly shaped by both host species identity and site (explaining 17.2% and 19.2% of the variation, respectively) (66). Here, we show that host identity and site characteristics act as selective forces shaping microbiome communities of *Sphagnum* spp. at the continental scale, each accounting for approximately 10% of the explained variation. Extreme environmental conditions in northern *Sphagnum*-dominated peatlands are relatively common and uniform across sites (67). Therefore, the consistent environment across large spatial scales may explain the more limited geographical and plant host effects on the observed community structure.

We define the core microbiome as taxa common to the microbial assemblages associated with a plant host that play an important role in host and ecosystem function (49). Although the core microbiome concept for plants is mainly defined by studies of model plants such as *Arabidopsis* (68, 69), a growing body of research on environmentally relevant plants indicates that years of coevolution led to the formation of a unique subset of the microbial community that correlates with plant health (44, 49, 70). However, the linkages between the core microbiome, plant health, and plant productivity remain unclear.

Core microbiomes of North American *Sphagnum* species are comprised of 12 common bacterial genera that contribute nearly 60% of the total relative microbial abundance. In corroboration of our work, investigations of two Alpine bogs in Austria revealed that approximately 50% of microbial communities are shared among sites, and community composition was not correlated with the degree of plant phylogeny (49). In a study of the leaves of 57 tree species in a neotropical forest in Panama, the core phyllosphere microbiome made up 73% of the total microbial abundance and was directly correlated with host growth, mortality, and function (70). In all cases, although representing a small minority of taxonomic diversity, shared taxa contributed to the majority of relative microbial abundance. While the implications for *Sphagnum* functional traits require further study, our results suggest that shared taxa play an important role in host and ecosystem functioning. Core microbiome analysis at the ASV and genus levels did not include any of the taxa known for their methanotrophic or diazotrophic capacities. However, analysis at the family level suggests that *Beijerinckiaceae* and *Methylacidiphilaceae* families collectively contribute almost 3% to the *Sphagnum* core microbiome. *Beijerinckiaceae* of the *Rhizobiales* contain psychrotolerant acidophilic bacteria with a versatile metabolism, including those capable of facultative and obligate methanotrophy (71, 72).

Mixotrophic methanotrophs of the *Methylacidiphilaceae* have evolved specific adaptations to overcome methane and nitrogen limitation. To meet energy and carbon demands, members of the *Methylacidiphilaceae* can grow heterotrophically on methane or autotrophically on hydrogen. However, optimal growth is achieved by combining these metabolic strategies. Hydrogen oxidation has particular importance for adaptation to methane and oxygen limitation (73, 74). In addition to methanotrophy, nitrogen fixation ability is a common feature of the *Beijerinckiaceae* and *Methylacidiphilaceae* (71–74). Thus, the diazotrophic and methanotrophic lifestyle of *Beijerinckiaceae* and *Methylacidiphilaceae* and their partnership with *Sphagnum* mosses likely contributed to their expansion across the North American continent.

We show that the *Sphagnum* core microbiome is dominated by moderately acidophilic chemo-organoheterotrophs known to utilize sugars, organic acids, and some polysaccharides as carbon and energy sources under oxic conditions (1). Core microbiome taxa consist mainly of members of the *Alphaproteobacteria* and *Acidobacteria*, which are known to be associated with *Sphagnum* and peat soils (*Acidocella*, *Granulicella*, *Acidisoma*, *Bryobacter*, *Acidisphaera*, *Phenylobacterium*, and WPS-2) (14, 26, 28, 36, 45–49, 75). Microbial cells in *Sphagnum* plants are thus far thought to be associated with dead hyaline cells, which comprise approximately 90% of the plant's volume (25) and serve as a hot spot of plant-microbe interactions. Hyaline cells may provide a favorable microhabitat with elevated pH and physical protection from bacterial predators (18, 76). Moreover, in contrast to the walls of cells that carry out photosynthesis in the *Sphagnum* gametophyte (chlorophyllose cells), polysaccharides such as arabinosylated β-galactans are enriched in hyaline cell walls (77). Thus, it follows that microbial taxa capable of utilizing these polysaccharides under acidic conditions will most likely dominate the microbial community. *Granulicella*, *Bryobacter*, and *Acidisoma* genera are aerobic chemo-organotrophic members of the *Sphagnum* core microbiome. These genera were initially isolated and characterized from *Sphagnum*-dominated peatlands. Moreover, these taxa are shown to degrade arabinose and other plant-related polysaccharides (78–80). The *Phenylobacterium* genus is an additional member of the *Sphagnum* core microbiome known for its capacity to degrade polyaromatic compounds (81).

Culture-independent methods frequently detect the candidate phylum WPS-2 in cold, acidic environments with high moss prevalence, where their abundances correlate well with abundances of methanotrophic and *Phenylobacterium* populations (14, 28, 47, 49, 82–85). Although not yet cultivated, moss-associated WPS-2 is believed to contain anoxygenic phototrophs with carbon fixation capacities (84). Anoxygenic phototrophic bacteria use light for energy along with sulfide, hydrogen, or ferrous iron as electron donors for carbon fixation (86). However, in peatlands, methane gas may represent an electron donor for anoxygenic phototrophy (87). Light-dependent carbon fixation, coupled with methane oxidation, has been reported for Rhodopseudomonas gelatinosa (*Bradyrhizobiaceae*) (88). Additionally, *Sphagnum*-associated diazotrophic members of the *Rhodopseudomonas* genus were shown to be resilient to multiyear warming stress (14) and probably play a role in peatland nitrogen and carbon budgets. Unfortunately, despite 50 years of research, no additional reports support the physiological link between methane oxidation and anoxygenic phototrophy. Although the phenotypes of WPS-2 taxa remain largely uncharacterized, their high relative abundance in *Sphagnum*-associated microbiomes and available draft genomes (84) motivates their successful isolation.

### Diazotrophy and its coupling to methanotrophy in the *Sphagnum* microbiome.

Overall, we show that known diazotrophs comprise a large portion of the *Sphagnum* microbiome community (up to 15% of sequence abundance), whereas methanotrophs are much less abundant (generally <0.2%) over large scales. Our results are corroborated by metagenomic investigations of peat soils and studies of *Sphagnum* microbiomes conducted over smaller scales. Surface peat from *Sphagnum*-dominated bogs, which contains an abundance of living *Sphagnum*, showed a high abundance and diversity of nitrogen fixation genes compared to other soil environments (14, 36, 49, 89). In agreement with our study, abundant diazotrophs were detected in the microbiomes of *S. fallax* and *S. magellanicum* in three Austrian bogs, while in contrast, methanotrophs comprised a much lower percentage of the overall community (27, 46).

Sequencing *nifH* amplicons over large scales corroborated SSU rRNA gene amplicon data to show that cyanobacteria of the *Nostocaceae* dominate *Sphagnum*-associated diazotrophic communities. While members of the *Nostoc*, *Fischerella*, and *Trichormus* genera comprised a large portion of the characterized diversity, *Nostoc* and *Fischerella* species contributed approximately 50% of expressed *nifH* genes. Previous work has yielded contradictory results on the potential significance of cyanobacteria in mediating nitrogen fixation in moss microbiomes. DNA-based approaches generally indicate that the cyanobacterial family *Nostocaceaea* (order *Nostocales*) predominates over *Sphagnum*-associated diazotrophs. Several studies, including those from the S1 bog studied here, suggest a central role for cyanobacterial diazotrophs in *Sphagnum* biomass accumulation (14, 33).

In contrast, other studies point to an essential role of the *Rhizobiales* within the *Alphaproteobacteria* in moss-associated nitrogen fixation (27, 36, 46, 48, 55, 56). Here, we show that while cyanobacteria of the genus *Nostoc* contributed ∼98% of the total *nifH* community in metagenomes from the S1 bog at the SPRUCE site, they comprised a minority (∼31%) of *nifH* transcripts in metatranscriptomes. Further, up to 40% of overall transcripts and 26% of *nifH* transcripts are taxonomically assigned to the known obligate methanotrophic genus *Methyloferula* (order *Rhizobiales*), despite their undetectable presence in SSU rRNA amplicons. Thus, we provide strong evidence that members of the *Rhizobiales* (and specifically *Methyloferula*), present at low abundance in *Sphagnum* microbiomes, represent keystone taxa that couple nitrogen fixation to methane oxidation. In agreement with our results, studies of wetlands in Florida and Georgia also revealed a substantial contribution of the rare biosphere to the mediation of the nitrogen cycle (90, 91).

Previous work on the physiological ecology of *Nostoc* supports our observations of apparent contradictions in its abundance and activity. Moss-associated *Nostoc* populations were shown to employ a “cheating” strategy whereby, despite high biomass, they exhibited low *nifH* expression levels (92). Although gene expression is not a direct indicator of fixation rates, it might indicate a limited contribution to the host's total nitrogen budget. Additionally, since the nitrogenase protein is irreversibly inhibited by oxygen, diazotrophs employ various strategies to separate nitrogen fixation from oxygenic photosynthesis (93). *Nostoc* is a genus of filamentous cyanobacteria that compartmentalize nitrogen fixation in specialized heterocystous cells (94). *Nostoc* colonization of bryophytes was shown to stimulate an increase in heterocyst density to approximately 25% to 45% of the total *Nostoc* cells (94). While *nifH* genes can be detected in all *Nostoc* cells, *nifH* expression is frequently restricted to heterocyst cells (93–96). Thus, for these reasons, *nifH* abundance at the DNA level may not serve as an accurate proxy for the nitrogen-fixing activity of *Nostoc* cells. Unfortunately, *Nostoc* was not included in our Chip-SIP analysis, and therefore its level of activity was not directly measured. Nevertheless, in previous SIP experiments with *Nostoc*-feather moss consortia, *Nostoc* was shown to fix nitrogen proportionally to carbon acquisition from the feather moss, and the feather moss incorporated *Nostoc*'s fixed nitrogen into its biomass (97, 98).

The primary focus of our Chip-SIP analysis was the hypothesis that methanotrophs of the *Sphagnum* microbiome couple the carbon and nitrogen cycles in peatlands. Previously, there was no direct evidence to support this dual capacity under natural conditions. Our Chip-SIP results demonstrate substantial incorporation of ^15^N_2_ and ^13^CH_4_ isotopes into SSU rRNA transcripts, indicating that members of the *Beijerinckiaceae* (which includes *Methyloferula*) and *Methylocystaceae* couple diazotrophy to methanotrophy under close to *in situ* conditions. To our knowledge, this study is the first to empirically couple nitrogen fixation with methane oxidation in the *Sphagnum* microbiome and provides a roadmap for further investigations.

Results from Chip-SIP experiments also corroborate our conclusion that members of the *Rhizobiales* represent keystone taxa in *Sphagnum* microbiomes. *Sphagnum*-associated microbial communities harbor diverse methanotrophic populations. The carbon fixed by those methanotrophic communities may provide up to one-third of plant tissue carbon (29, 32). Most methanotrophs observed in *Sphagnum* tissues belong to the *Alphaproteobacteria* (29, 32, 36, 48, 56–59). A subset of these methanotrophs have the genetic potential for nitrogen fixation (i.e., type II *Methylosinus* spp., *Methylocystis* spp., *Methyloferula* spp.) (99, 100). In this study, SSU rRNA analysis indicates that the genus *Methylocystis* dominates *Sphagnum*-associated methanotrophic communities across the North American continent, including the SPRUCE site. Stable isotope probing experiments have confirmed the ability of these genera to mediate methanotrophy in *Sphagnum* microbiomes (58, 59, 101–103).

### Implications for biogeochemical cycles in peatlands.

In this study, multiple lines of evidence indicate that members of the *Rhizobiales* play a key role in coupling nitrogen fixation to methanotrophy. Our results corroborate biogeochemical field data, which showed a coupling of nitrogen fixation and methane oxidation in *Sphagnum*-dominated peatlands (34). The fact that plant communities, especially mosses, thrive in nutrient-poor peatland ecosystems is a well-established paradox. By definition, external nutrient inputs to ombrotrophic bogs (e.g., the SPRUCE site) are limited to deposition from rain or snow. Consequently, the nitrogen demand from plants in *Sphagnum*-dominated bogs far exceeds inputs from precipitation or internal cycling (34–36). These observations have led others to suggest that diazotrophic methanotrophs may be responsible for the “unaccounted nitrogen input” in peatlands, thereby providing a “missing link” in the biogeochemical cycles of nitrogen and carbon (63). Under this scenario, there is an active exchange of compounds between methanotrophs, diazotrophs, and *Sphagnum*. However, the specific mechanisms of exchange and ecological relevance of this coupling in *Sphagnum* microbiomes has been unresolved. Here, we show that an obligate methanotroph, *Methyloferula*, which relies on methane oxidation for energy generation, is highly active in *Sphagnum* microbiomes from an ombrotrophic bog at the SPRUCE site. Further, we show that the *Beijerinckiaceae*, which include the genus *Methyloferul*a, closely couple diazotrophy to methanotrophy in dual-isotope tracer experiments. Although undetectable in amplicon sequence libraries, *Methyloferula* comprised approximately 0.2% of prokaryotic genes and transcripts in our metagenomes and metatranscriptomes, respectively. Thus, our results suggest that diazotrophic methanotrophs of the rare biosphere play a keystone role in coupling of the carbon and nitrogen cycles in peatlands. The significance of diazotrophic methanotrophs, and *Methyloferula* in particular, could be confirmed with more highly resolved *in situ* physiological approaches such as nanoscale secondary ion mass spectrometry (nanoSIMS) (104, 105).

## MATERIALS AND METHODS

### Sample collection.

During the growing season in 2014, 2015, and 2016, over 250 *Sphagnum* microbiome samples were collected from peatlands across 5 states and 17 bog/fen sites, including 18 *Sphagnum* genotypes (see Table S1 at https://zenodo.org/record/5786378). Nondestructive plant taxonomic identification was performed *in situ* by visual inspection at collection. Living *Sphagnum* plants were collected using sterile tweezers and scissors. The collected plants were cleaned to remove unrelated plant debris and frozen on dry ice. Frozen samples were shipped overnight to the lab and stored at −80°C until analysis.

### Total DNA extraction, PCR, and amplicon sequencing.

Total DNA was extracted as previously described (14) (see Text S1 in the supplemental material). The V4 variable region of small subunit (SSU) rRNA genes and the conserved fragment of dinitrogenase reductase subunit (*nifH*) genes were amplified with 515F/806R and IGK3/DVV primers, respectively, and sequenced on the Illumina platform at the University of Illinois at Chicago (14, 91) (Text S1; see Table S2 at https://zenodo.org/record/5786378).

### Amplicon data processing and statistical analyses.

Raw fastq files were processed as previously described (14, 91) (Text S1). The final high-quality data sets contained 8,049,198 SSU rRNA gene sequences grouped into 12,044 unique ASVs and represent 246 samples (median of 31,569 reads/sample). Similarly, 830,598 *nifH* gene sequences clustered into 8,934 unique ASVs and represent 195 samples (median of 3,657 reads/sample). High-quality sequence data sets were normalized by cumulative sum scaling (CSS), and major variance components of beta diversity were determined using nonmetric multidimensional scaling (NMDS) of Bray-Curtis and weighted UniFrac distance matrices. Significant differences in beta diversity were analyzed by a PERMANOVA test on weighted UniFrac distance metrics with 1,000 permutations. The ordination and statistical analyses were performed in phyloseq and vegan R packages (106, 107).

### Omics sequencing.

Triplicate individual plants of *Sphagnum fallax* and *Sphagnum magellanicum* were collected in August 2015 from the SPRUCE experimental site at the S1 bog in the Marcell Experimental Forest (http://mnspruce.ornl.gov). One gram of plant tissue was ground in liquid nitrogen and used for nucleic acid extractions (Text S1). The absence of DNA contamination in the RNA extracts was confirmed by a PCR with universal bacterial 16S rRNA primers 515F and 806R (see Table S2 at https://zenodo.org/record/5786378). The nucleic acid extracts were shipped to the Joint Genome Institute (JGI; https://jgi.doe.gov/) for the metagenomic and metatranscriptomic library construction and sequencing (Text S1).

### Illumina data assembly and annotation.

For the metagenome and metatranscriptome contig-based analysis, the quality trimmed reads were coassembled into approximately 3.4 million contigs (Text S1). We calculated the percentage of the reads recruited by contigs for each omics library using Bowtie2 (108) to estimate how well the assembly represented the original raw data. The protein-encoding regions known as open reading frames (ORFs) were predicted with MetaProdigal (109). Predicted ORFs were assigned to KEGG databases by running a KofamScan script against HMM models of KEGG orthologs (KOs) (110). The contigs and ORF taxonomy were assigned using the Kraken2 classifier (111) and GTDB v.85 databases (https://github.com/Ecogenomics/GtdbTk). Finally, high-quality reads were mapped back to each contig and ORFs with Bowtie2 (108), and RPKM counts (reads per kilobase million) were calculated to estimate the abundances of each contig and ORF.

### Microarray stable isotope probing (Chip-SIP)—linking phylogeny with function.

The identity of active diazotrophs and methanotrophs was determined from ^15^N and ^13^C isotope incorporation into SSU rRNA transcripts using the Chip-SIP approach (60, 61) (see Text S1 in the supplemental material). Briefly, 10 independent replicates of *Sphagnum* samples were collected from the peat surface during the growing season of 2015 from the SPRUCE experimental site. Ten grams was placed into a 125-mL gas-tight serum bottle, and 50 mL of headspace gas was replaced with 40 mL ^15^N_2_ and 10 mL ^13^CH_4_ (Cambridge Isotope Laboratories, Andover, MA, USA). Treatments were incubated at 20°C under natural light conditions, and duplicates of total RNA were extracted after 12 days of incubation using the MOBIO PowerSoil kit (Qiagen, Carlsbad, CA, USA). Extracted RNA samples were fluorescently labeled and hybridized to a phylogenetic probe microarray (62) (Text S1). A custom phylogenetic probe array was designed based on our sequence data set from the SPRUCE site (9, 59, 89, 112) and NCBI RefSeq database. This set included 4,072 phylogenetic probes targeting 392 SSU rRNA gene probes from 45 families designed to target known bacterial and archaeal diazotrophs and/or methanotrophs, not including cyanobacteria. Relative isotope incorporation was calculated as the ratio between isotopic and fluorescent signals (hybridization-corrected enrichment [HCE]). Microbial taxa were considered metabolically active if HCE was significantly different from zero (60, 61) (Text S1). We constructed a bipartite network to visualize taxa that showed significant enrichment (*P* < 0.05 after false discovery rate *P* value adjustment) by one or more isotopes in at least one sample. Note that these data are relatively quantitative and represent average relative isotope incorporation across samples where isotope incorporation was detected. The network reconstruction was done with the R package igraph (113).

### Data availability.

The raw amplicon sequences were deposited in the BioProject database under accession numbers PRJNA656910 (SSU rRNA) and PRJNA656922 (*nifH*). The raw metagenomic and metatranscriptomic sequences are publicly available under accession number Gs0118677.
