# Supplementary figures and images for: Defining the Sphagnum Core Microbiome across the North American Continent Reveals a Central Role for Diazotrophic Methanotrophs in the Nitrogen and Carbon Cycles of Boreal Peatland Ecosystems
Source: mBio. 2022 Feb 22;13(1):e03714-21. doi: 10.1128/mbio.03714-21 (PMC8863050; doi:10.1128/mbio.03714-21)

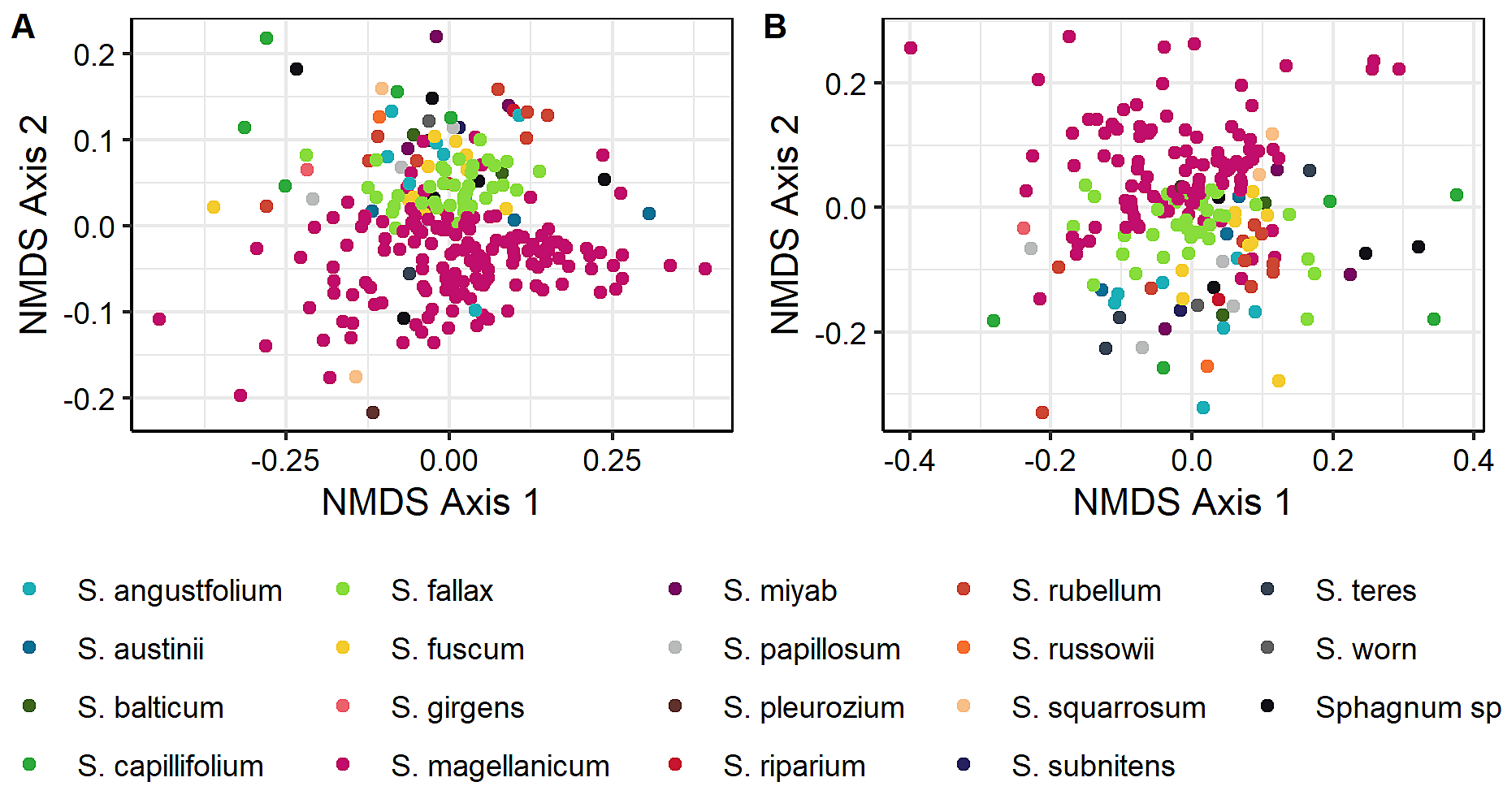

Supplement: FIG S1 [file mbio.03714-21-sf001.tif]

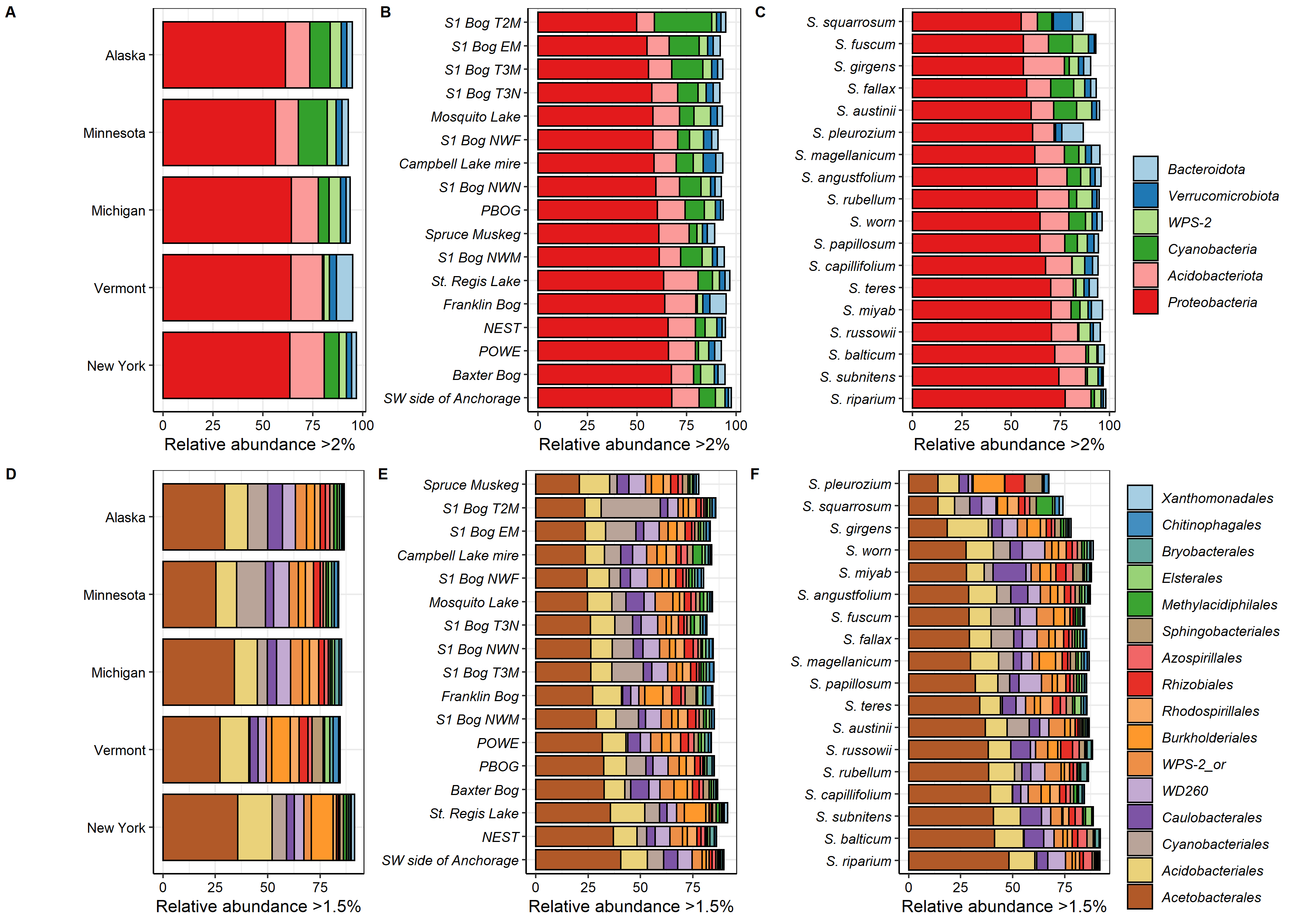

Supplement: FIG S2 [file mbio.03714-21-sf002.tif]

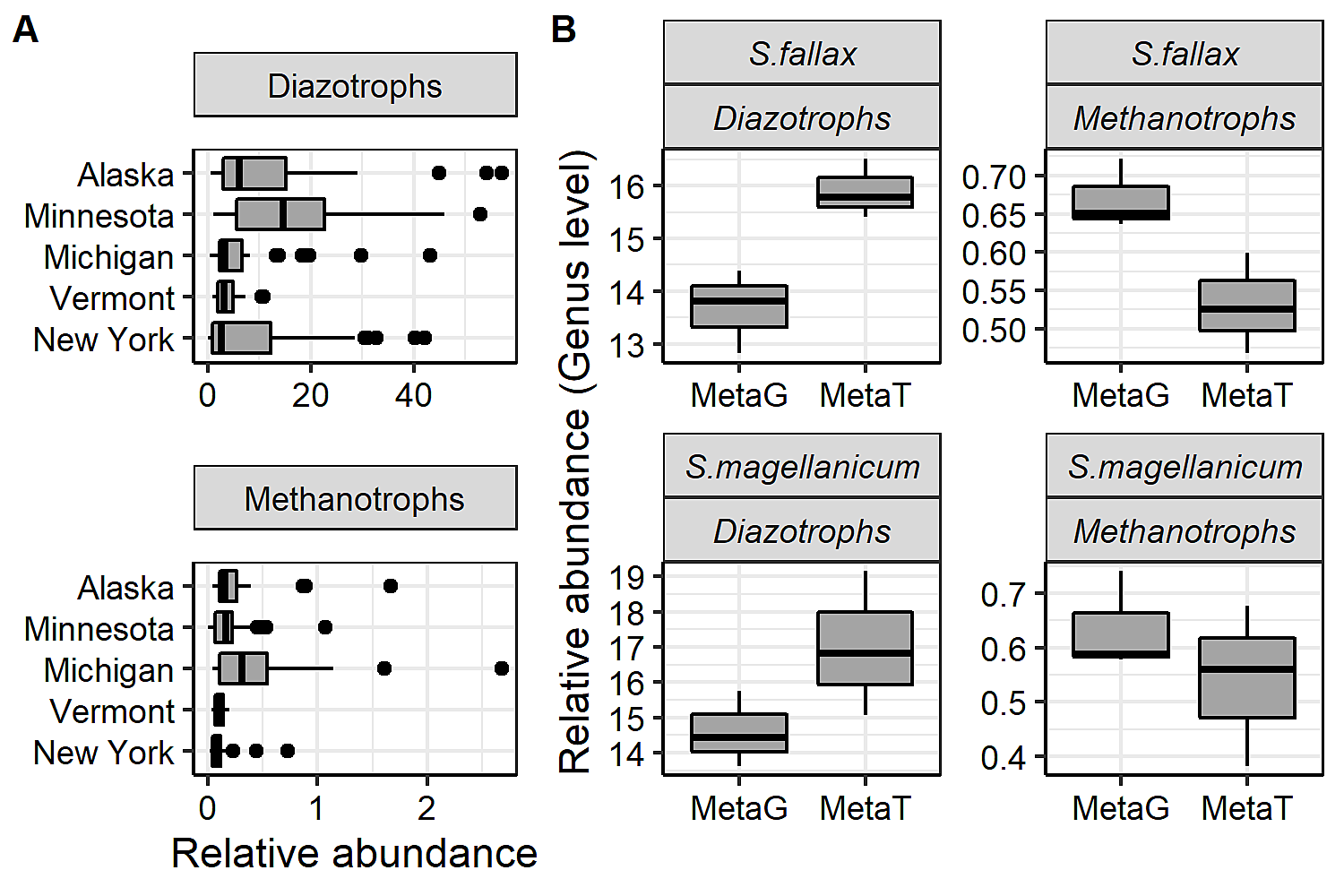

Supplement: FIG S3 [file mbio.03714-21-sf003.tif]

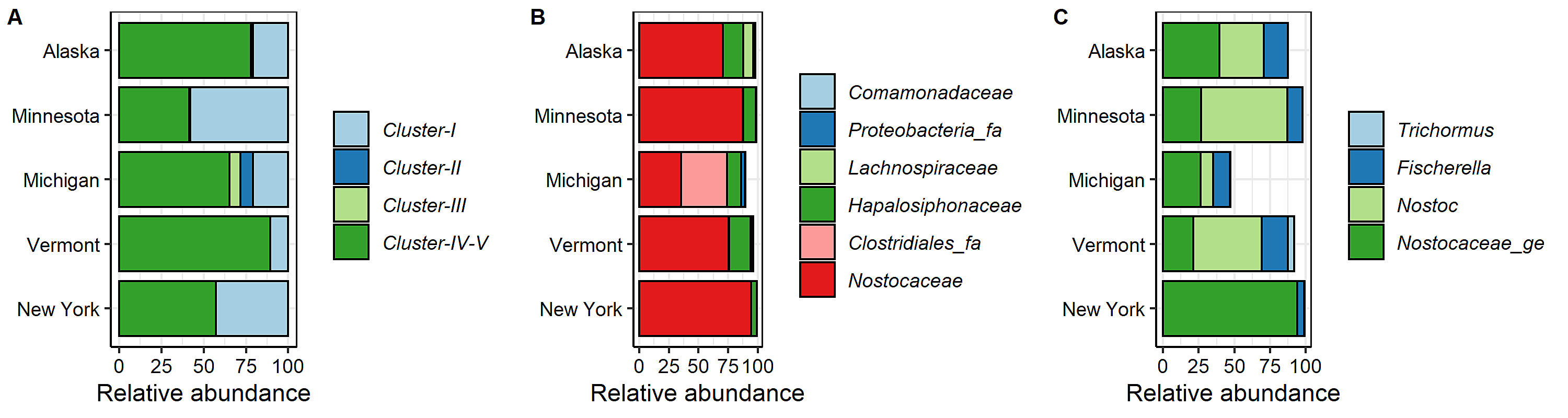

Supplement: FIG S4 [file mbio.03714-21-sf004.tif]

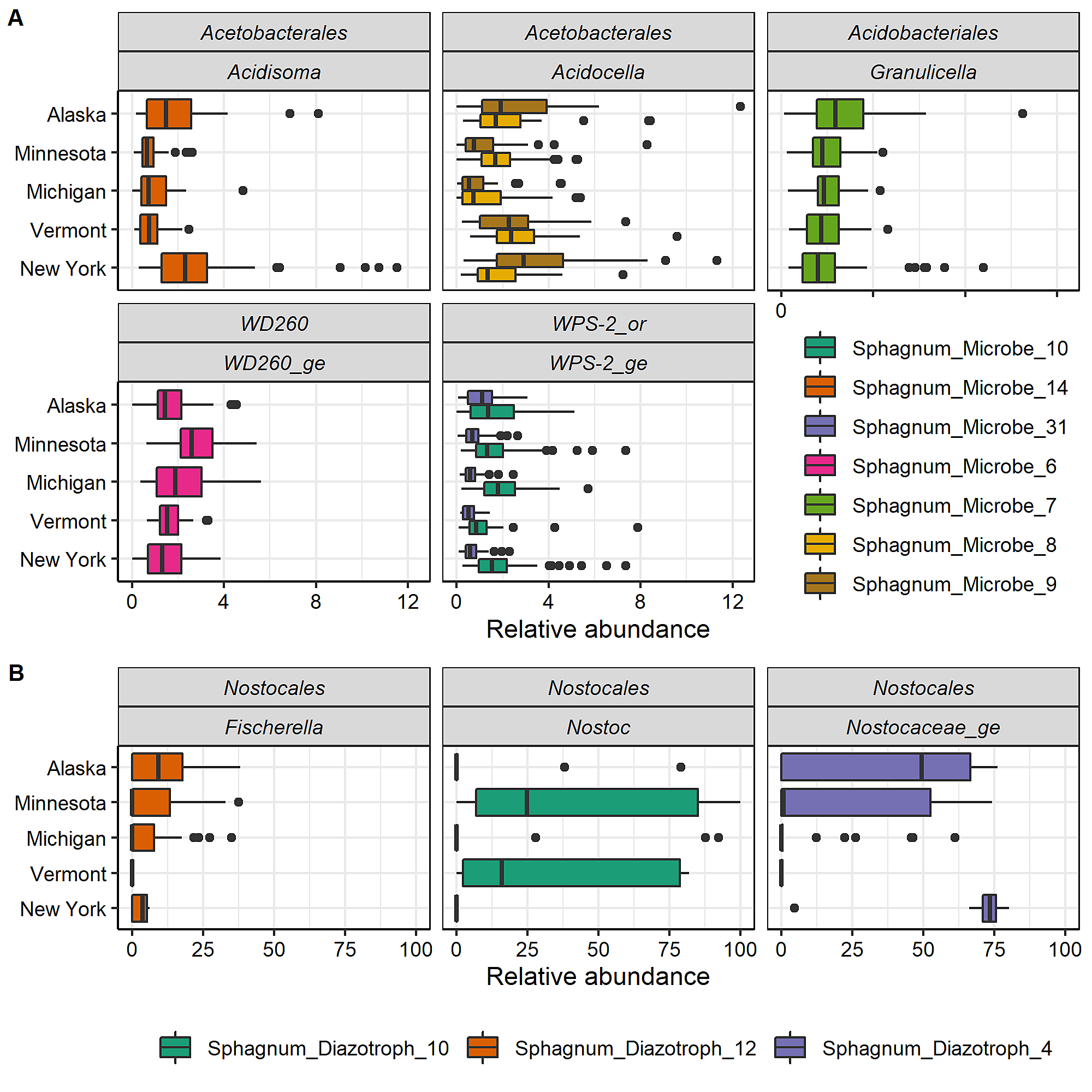

Supplement: FIG S5 [file mbio.03714-21-sf005.tif]

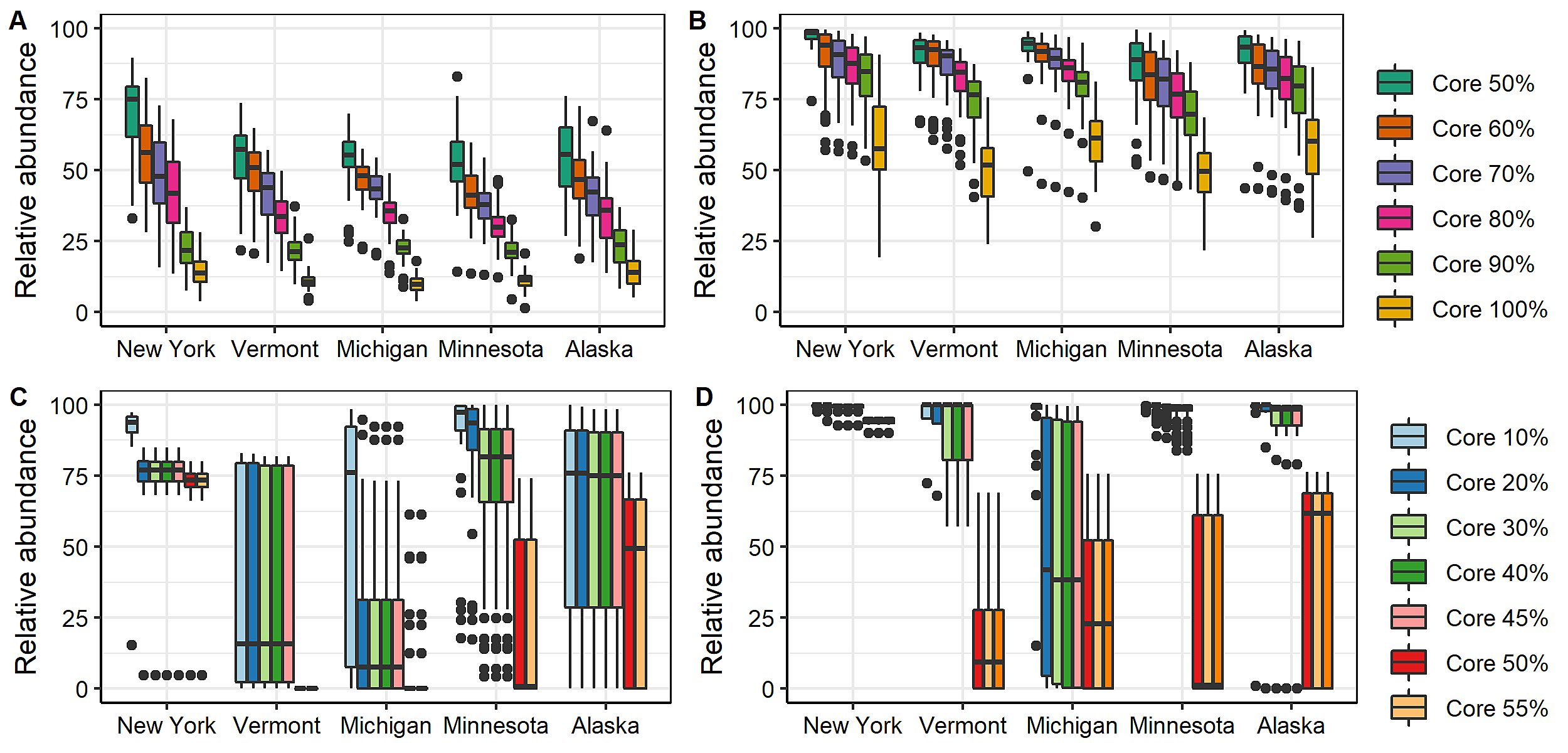

Supplement: FIG S6 [file mbio.03714-21-sf006.tif]

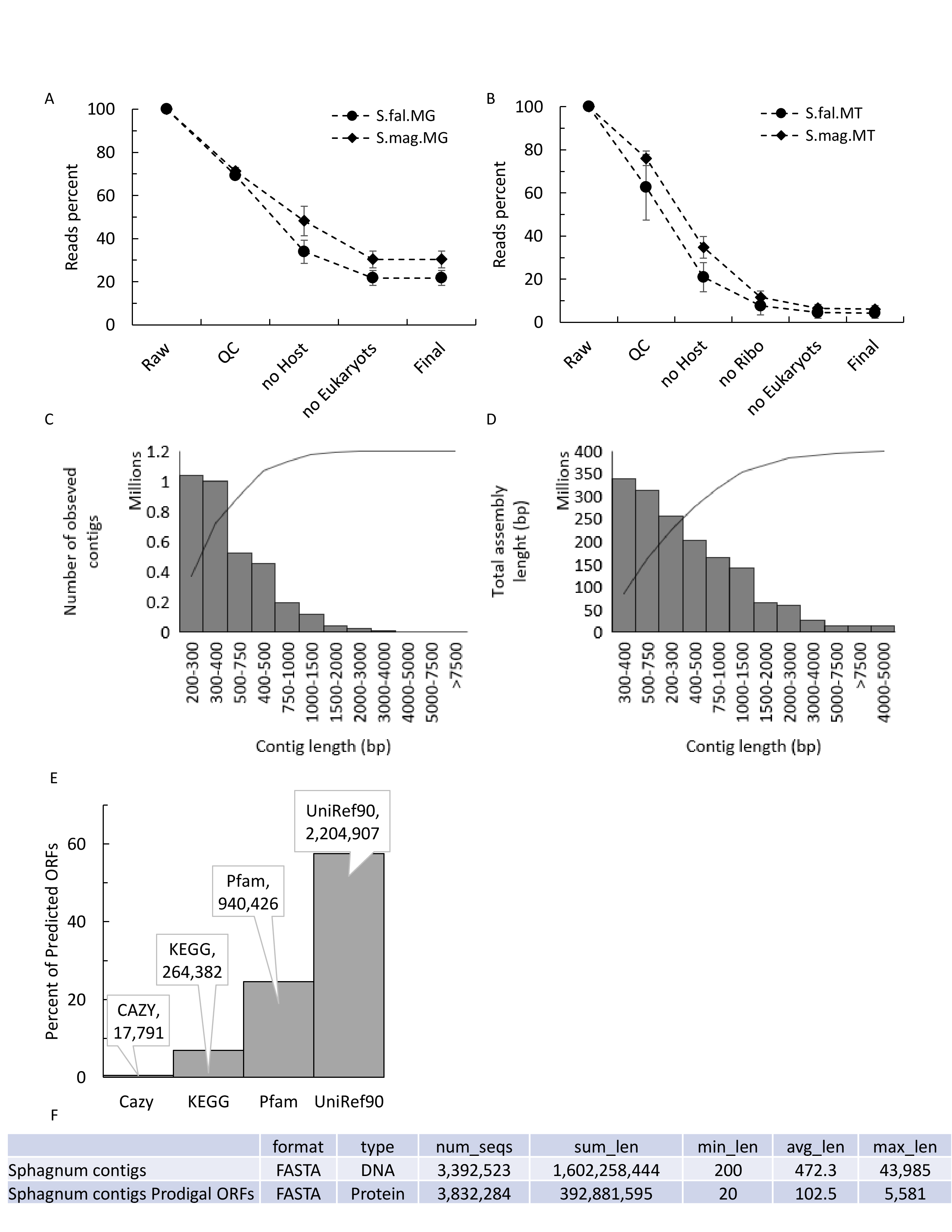

Supplement: FIG S7 [file mbio.03714-21-sf007.tif]

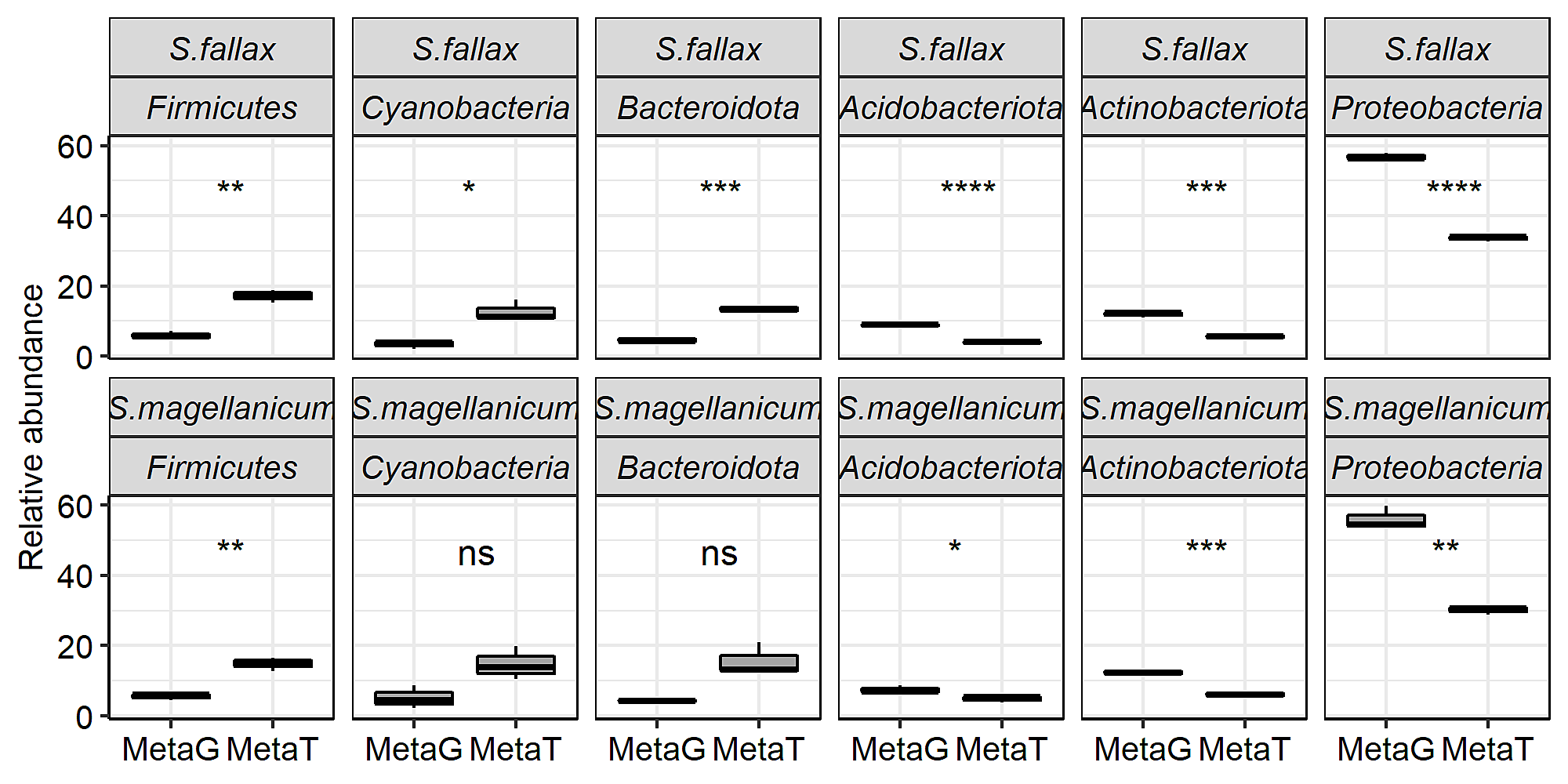

Supplement: FIG S8 [file mbio.03714-21-sf008.tif]

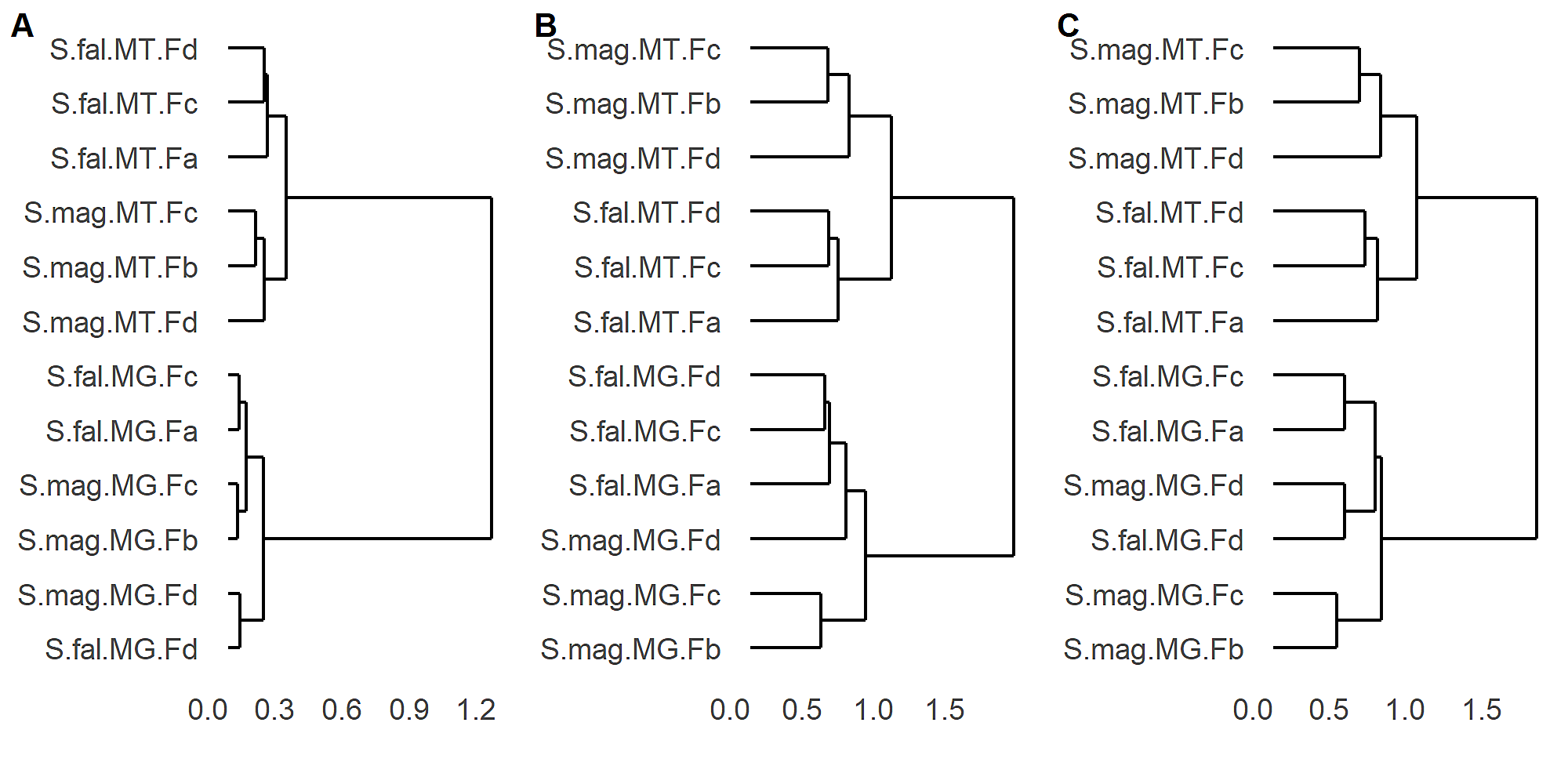

Supplement: FIG S9 [file mbio.03714-21-sf009.tif]
