# Supplementary material for: Defining the Sphagnum Core Microbiome across the North American Continent Reveals a Central Role for Diazotrophic Methanotrophs in the Nitrogen and Carbon Cycles of Boreal Peatland Ecosystems
Source: mBio. 2022 Feb 22;13(1):e03714-21. doi: 10.1128/mbio.03714-21 (PMC8863050; doi:10.1128/mbio.03714-21)
Supplement: TEXT S1 [file mbio.03714-21-s0001.docx]

**­­­Supplementary methods****­­­**

**Defining the *Sphagnum* core microbiome across the North American continent revealed a central role of diazotrophic-methanotrophs in nitrogen and carbon cycles of boreal peatland ecosystems.**

Max Kolton^a*^, David J. Weston^b^, Xavier Mayali^c^, Peter K. Weber^c^, Karis J. McFarlane^c^, Jennifer Pett-Ridge^c^, Mark M. Somoza^jk^, Jory Lietard^j^, Jennifer B. Glass^a^, Erik A. Lilleskov^d^, A. Jonathan Shaw^e^, Susannah Tringe^f^, Paul J. Hanson^b^, Joel E. Kostka^a#^

^a^School of Biology and School of Earth & Atmospheric Sciences, Georgia Institute of Technology, Atlanta, Georgia, USA

^b^Biosciences Division, Oak Ridge National Lab, Oak Ridge, Tennessee, USA

^c^Physical and Life Sciences Directorate, Lawrence Livermore National Laboratory, Livermore, California, USA

^d^Northern Research Station, USDA Forest Service, Houghton, Michigan, USA

^e^Biology Department, Duke University, Durham, North Carolina, USA

^f^DOE Joint Genome Institute and Environmental Genomics and Systems Biology Division, Lawrence Berkeley National Laboratory, Berkeley, California, USA

^j^Department of Inorganic Chemistry, University of Vienna, Vienna, Austria

^k^Leibniz Institute for Food Systems Biology and Chair of Food Chemistry and Molecular and Sensory Science, Technical University of Munich, Freising, Germany

Running Head: Methanotrophic-diazotrophy of *Sphagnum* microbiome

#Address correspondence to Joel E. Kostka [Joel.Kostka@biology.gatech.edu](mailto:Joel.Kostka@biology.gatech.edu)

*Present address: French Associates Institute for Agriculture and Biotechnology of Drylands, Ben-Gurion University of the Negev, Beer Sheva, Israel

1. Sample preparation, DNA extraction, PCR, and sequencing
2. Amplicon data processing
3. Metagenomic and metatranscriptomic nucleic acids extraction and sequencing
4. Metagenomic and metatranscriptomic quality control
5. Illumina data assembly and annotation
6. Chip-stable isotope probing (Chip-SIP), Linking phylogeny with function
7. **Sample preparation, DNA extraction, PCR, and sequencing**

Samples were prepared, and total DNA was extracted using the DNeasy PowerSoil kit (Qiagen, Carlsbad, USA) as described previously (1). Extracted DNA was quantified with the Qubit HS assay (Invitrogen, Carlsbad, USA). Five ng of the DNA template was used to determine the diversity and composition of *Sphagnum*‐associated prokaryotic and diazotrophic communities. The V4 variable region of small subunit (SSU) rRNA genes and the conserved fragment of dinitrogenase reductase subunit (*nifH*) genes were amplified with 515F/806R and IGK3/DVV primer pairs, respectively, as described previously ((1, 2), Table S2). The SSU rRNA and *nifH* PCR products were tagged with unique Fluidigm barcodes (Fluidigm Corporation, San Francisco, USA) and sequenced on the Illumina platform at the Research Resources Center in the University of Illinois at Chicago according to standard protocols. The SSU rRNA and *nifH* gene raw amplicon sequences were deposited in the BioProject database (<http://ncbi.nlm.nih.gov/bioproject>) under accessions PRJNA656910 and PRJNA656922, respectively.

1. **Amplicon data processing**

Prior to the analysis, the forward and reverse primers were removed with Cutadapt (3). The primer-trimmed sequences were quality-filtered, dereplicated, and merged into error-corrected amplicon sequence variants (ASVs) using the DADA2 R package (4). ASVs that exceeded the expected size range of 251–255 bp or 330–360 bp for SSU rRNA V4 variable regions and dinitrogenase reductase subunit (*nifH*) gene fragments, respectively, were excluded. Chimera sequences were identified and removed using the removeBimeraDenovo function of the DADA2 package (4). The resulting high-quality sequences were aligned with the SILVA SSU rRNA (release 138) or *nifH* reference alignments (5, 6), and an approximate maximum likelihood tree was constructed using FastTree v.2.1 (7). Taxonomies were assigned to each ASV using the RDP Naïve Bayesian Classifier (8) with SILVA SSU rRNA (release 138) or *nifH* reference databases (5, 6) as implemented in Mothur v.1.40 software (9) with a minimum confidence threshold of 50%. Sequences classified as ‘eukaryotes’, ‘chloroplast’, ‘mitochondria’ or that did not match any taxonomic class were excluded from the dataset. The resultant datasets were filtered to remove low coverage or overrepresenting libraries. The samples having lower than 15,000 or greater than 65,000 of prokaryotic reads were removed. Similarly, samples having diazotrophic reads lower than 1,000 or greater than 12,000 were excluded. Finally, ASVs that appeared only once in the dataset and/or had less than 15 counts were removed from the final datasets. After discarding rare ASVs, a total of 8,049,198 SSU rRNA gene sequences distributed across 246 samples (median of 31,569 reads per sample) and 12,044 unique SSU rRNA ASVs remain for downstream analysis. Additionally, a total of 830,598 *nifH* sequences distributed across 195 samples (median of 3,657 reads per sample) and 8,934 unique *nifH* ASV were retained for community analysis. High-quality sequence datasets were normalized by cumulative sum scaling (CSS), and major variance components of beta diversity were determined using non-metric multidimensional scaling (nMDS) of Bray-Curtis and weighted unifrac distance matrices. Significant differences in beta diversity were analyzed by a PERMANOVA test on weighted Unifrac distance metrics with 1,000 permutations.

1. **Metagenomic and metatranscriptomic nucleic acids extraction and sequencing.**

Triplicate individual plants of *Sphagnum fallax* and *Sphagnum magellanicum* were collected in August 2015 from the SPRUCE experimental site at the S1 bog in the Marcell Experimental Forest (<http://mnspruce.ornl.gov>) and frozen on dry ice for transport back to the laboratory. One gram of plant tissue was ground in liquid nitrogen and used for RNA and DNA extraction. Total DNA was extracted using the PowerPlant DNA isolation kit following the manufacturer’s protocol (Qiagen, Carlsbad, USA). Total RNA was isolated using the PowerPlant RNA isolation kit with DNase in the same manner (Qiagen, Carlsbad, USA). The absence of DNA contamination in the RNA extracts was confirmed by running a PCR using universal bacterial 16S rRNA primers 515F and 806R (Table S2). A detailed RNA extraction protocol was previously described (10). Total DNA and DNA-free RNA samples were shipped on dry ice to the Joint Genome Institute (JGI, <https://jgi.doe.gov/>) for metagenomic and metatranscriptomic library construction and sequencing. Before the metatranscriptome library construction, ribosomal rRNA was subtracted using the Ribo-Zero rRNA removal kit (Illumina, San Diego, USA). Finally, the TruSeq kit was applied to construct paired-end metagenomic and metatranscriptome libraries from DNA and rRNA-depleted RNA samples, respectively. The resultant libraries were sequenced on the Illumina HiSeq 2500 sequencer using a pair-end 150-bp flow cell. The raw metagenomic and metatranscriptomic sequences are publicly available on the JGI website under accession number Gs0118677 (<https://gold.jgi.doe.gov/biosamples?Study.GOLD%20Study%20ID=Gs0118677>).

1. **Metagenomic and metatranscriptomic quality control**

Illumina paired-end 150 bp metagenomic and metatranscriptomic raw reads were trimmed from adapters, sequencing artifacts, and poor-quality bases with BBDuk (<http://jgi.doe.gov/data-and-tools/bb-tools/>), setting a minimum Phred-like quality of 25, and a minimum length of 100 and 75 nucleotides for metagenomic and metatranscriptomic reads, respectively. The quality of raw and trimmed reads were inspected using the FASTQC software (<https://www.bioinformatics.babraham.ac.uk/projects/fastqc/>, Babraham Bioinformatics, Cambridge, UK). *S. magellanicum*, *S. fallax*, and human-related reads were removed from the metagenome and metatranscriptomic samples to reduce host contamination. Briefly, trimmed reads were aligned to the *S. magellanicum* (<https://gold.jgi.doe.gov/projects?id=Gp0060578>), *S. fallax* (<https://phytozome-next.jgi.doe.gov/info/Sfallax_v0_5>), and human genomes (GRCh38) using Bowtie2 (11) with options –very-sensitive-local and -k 1. Host reads-free FASTQ files were generated from reads which did not align (carrying SAM flag -f 12) using samtools (12). To improve assembly quality, residual ribosomal RNA (rRNA) reads were filtered out from metatranscriptomic libraries using SortMeRNA (13). Subsequently, reads were classified with Kraken2 (14) using a custom Kraken database containing NCBI non-redundant nucleotide database and all archaeal, bacterial, fungal, viral, and plant RefSeq genomes. Finally, eukaryotic and viral reads were excluded from the reads collection (Fig. S6A, B, Table S6).

In total, 189 and 134 Gbp were generated from the six metagenomic and six metatranscriptomic libraries, respectively. After quality filtering, approximately 70% of the metagenomic and 45% of the metatranscriptomic reads were retained for downstream analysis. Following quality control, *Sphagnum* genome and other eukaryotic reads were excluded. Finally, ribosomal RNA sequences were removed from the metatranscriptomic libraries. This approach resulted in 47.6 and 2.3 Gbp of high quality metagenomic and metatranscriptomic reads, respectively (Fig. S6, Table S6). Reads were co-assembled into 3.4 million contigs with a total length of 1.6 Gbp, encoding approximately 3.8 million predicted proteins (Fig. S6). The resulting assembly recruited approximately 40 and 80% of the metagenomic and metatranscriptomic reads, respectively.

1. **Illumina data assembly and annotation.**

The preprocessed reads were normalized and error-corrected with the bbnorm function of BBMap (<http://jgi.doe.gov/data-and-tools/bb-tools/>) with an average target coverage of 100x, and metagenomic samples were co-assembled using MEGAHIT (15). Subsequently, the metatranscriptomic reads were mapped to the contigs with Bowtie2 (11). Unmapped reads were extracted and co-assembled with Trinity (16). Furthermore, the contigs recovered from both assemblies were pooled to create consensus assembly (17, 18). Initially, contigs shorter than 200bp were discharged. Then, the contigs shorter than 2 kbp were subtracted and merged with CAP3 assembler (19), and the resultant contigs were used for the secondary assembly. To reduce the computational time required for secondary assembly, the contigs dataset was reduced by combining contigs with ≥99% semi-global identity with cd-hit-est (20). Finally, contigs were integrated using Minimus2 with -D OVERLAP=100 MINID=95 parameters (21). This approach generated a consensus co-assembly containing approximately 3.4 million contigs used for downstream analysis. We calculated the percent of the reads recruited by contigs for each omics library using Bowtie2 (11) to estimate how well the assembly represented the original raw data (11).The protein encoded regions known as open reading frames (ORFs) in the final contigs were predicted with Meta Prodigal (22). The predicted ORFs sequences were functionally annotated against the Pfam (23) and Uniref90 (24) databases using DIAMOND (25). Additionally, predicted ORFs were assigned to KEGG databases by running KofamScan script against HMM models of KEGG Orthologs (KOs) (26). The taxonomy of the final contigs and predicted ORFs were assigned using Kraken2 classifier (14) and GTDB v.85 databases (<https://github.com/Ecogenomics/GtdbTk>). Finally, high-quality reads were mapped back to each contig and ORFs with Bowtie2 (11), and RPM (Reads Per Kilobase Million) counts were calculated to estimate each contig and ORF abundances.

1. **Chip-stable isotope probing (Chip-SIP), Linking phylogeny with function.**

The identity of the active diazotrophs and methanotrophs was determined by incorporating ^15^N and ^13^C isotopes from ^15^N_2_ and ^13^CH_4_ into SSU rRNA transcripts using the Chip-SIP approach (27, 28). Four and six independent replicates of Sphagnum samples were collected from the peat surface in June and August 2015, respectively, from the SPRUCE experimental site at the S1 bog in the Marcell Experimental Forest (<http://mnspruce.ornl.gov>). Ten grams were placed into a 125 ml gas-tight serum bottle, and 50 ml of headspaces gas were replaced with 40 ml ^15^N_2_ and 10 ml ^13^CH_4_ (Cambridge Isotope Laboratories, Andover, USA). Treatments were incubated at 20℃ in a plant growth chamber under natural light conditions. After 5, 12, and 19 days of incubation, samples were destructively collected for isotope ratio mass spectrometry (IRMS) to determine bulk ^15^N and ^13^C incorporation. Subsequently, total RNA was extracted from the 12-day incubation samples using the RNEasy RNA extraction kit according to manufacturer protocol (Qiagen, Carlsbad, USA) and processed for Chip-SIP analysis. Extracted RNA samples were fluorescently labeled and hybridized to a phylogenetic probe microarray synthesized at the University of Vienna, as previously described (29). A custom phylogenetic probe set was constructed with our sequence dataset from the SPRUCE site (30-33) and NCBI RefSeq database. This set included 4,072 phylogenetic probes targeting 392 SSU rRNA gene probes from 45 families designed to target bacterial and archaeal groups hypothesized to be involved in nitrogen fixation and methane oxidation. We note that cyanobacteria were not targeted in this dataset. These taxa were chosen based on Illumina 16S rRNA sequences, but full 16S rRNA gene sequences from SILVA v. 123 were used for probe design, so the probes spanned the entire length of the 16S rRNA gene. For each taxon, 25 bp probes were designed to target sequences >99% similar to a representative ASV sequence using ARB (34), and probes were removed if they were not taxonomically specific, formed hairpins, or had high G+C content or too many G and C in a row (homopolymer runs).

Non-fluorescently labeled (but isotope-labeled) RNA samples were hybridized on a duplicate array to the fluorescently-labeled samples, but the array was synthesized on an ITO (indium tin oxide) coated slide functionalized to enable DNA synthesis (superepoxy2 coating, Arrayit, Sunnyvale, CA, USA). Hybridized arrays were analyzed with the LLNL NanoSIMS 50 with a 2 pA cesium ion beam, collecting ^12^C^14^N^-^, ^13^C^14^N^-^, ^12^C^15^N^-^, and ^13^C^15^N^-^ in magnetic switching mode. Mass resolving power was set at 10,000 with aperture slit 3 and entrance slit 5 to resolve ^13^C^14^N^-^ from ^11^B^16^O^-^. Ion images were stitched together and processed to generate isotopic ratios, and regions of interests (ROIs) of the individual probe spots extracted with the L’IMAGE software (L. Nittler, Carnegie Institution of Washington). For a subset of the experiments, we examined the ability to detect dual-labeling with ^13^C^15^N/^12^C^15^N and ^13^C^15^N/^13^C^14^N (mass 28 over mass 27), rather than the traditional ^13^C^14^N/^12^C^14^N and ^12^C^15^N/^12^C^14^N (mass 27 over mass 26). Since many of the taxa were labeled with both ^13^C and ^15^N, measuring mass 28 was more sensitive in detecting isotope incorporation due to the lower background. Relative isotope incorporation was calculated as the ratio between isotopic and fluorescent signals (hybridization-corrected enrichment, HCE). Microbial taxa were considered metabolically active if HCE was significantly different from zero (27, 28). For each sample, all positive HCE values were normalized to the highest value for that sample to compare among samples. We constructed a bipartite network to visualize taxa that showed significant enrichment (p < 0.05 after false discovery rate p-value adjustment) by one or more isotopes. The network reconstruction was done by using the R package igraph (35).

**REFERENCES**

1. Carrell AA, Kolton M, Glass JB, Pelletier DA, Warren MJ, Kostka JE, Iversen CM, Hanson PJ, Weston DJ. 2019. Experimental warming alters the community composition, diversity, and N_2_ fixation activity of peat moss (*Sphagnum fallax*) microbiomes. Global Change Biology 25:2993-3004.

2. Kolton M, Rolando JL, Kostka JE. 2020. Elucidation of the rhizosphere microbiome linked to *Spartina alterniflora* phenotype in a salt marsh on Skidaway Island, Georgia, USA. Fems Microbiology Ecology 96:fiaa026.

3. Martin M. 2011. Cutadapt removes adapter sequences from high-throughput sequencing reads. 2011 17:3.

4. Callahan BJ, McMurdie PJ, Rosen MJ, Han AW, Johnson AJA, Holmes SP. 2016. DADA2: High-resolution sample inference from Illumina amplicon data. Nature Methods 13:581-581.

5. Quast C, Pruesse E, Yilmaz P, Gerken J, Schweer T, Yarza P, Peplies J, Glockner FO. 2013. The SILVA ribosomal RNA gene database project: improved data processing and web-based tools. Nucleic Acids Research 41:D590-D596.

6. Gaby JC, Rishishwar L, Valderrama-Aguirre LC, Green SJ, Valderrama-Aguirre A, Jordan IK, Kostka JE. 2018. Diazotroph community characterization via a high-throughput *nifH* amplicon sequencing and analysis pipeline. Applied and Environmental Microbiology 84:e01512-17.

7. Price MN, Dehal PS, Arkin AP. 2009. FastTree: Computing large minimum evolution trees with profiles instead of a distance matrix. Molecular Biology and Evolution 26:1641-1650.

8. Wang Q, Garrity GM, Tiedje JM, Cole JR. 2007. Naive Bayesian classifier for rapid assignment of rRNA sequences into the new bacterial taxonomy. Applied and Environmental Microbiology 73:5261-5267.

9. Schloss PD, Westcott SL, Ryabin T, Hall JR, Hartmann M, Hollister EB, Lesniewski RA, Oakley BB, Parks DH, Robinson CJ, Sahl JW, Stres B, Thallinger GG, Van Horn DJ, Weber CF. 2009. Introducing mothur: Open-source, platform-independent, community-supported software for describing and comparing microbial communities. Applied and Environmental Microbiology 75:7537-7541.

10. Stough JMA, Kolton M, Kostka JE, Weston DJ, Pelletier DA, Wilhelm SW. 2018. Diversity of active viral infections within the *Sphagnum* microbiome. Applied and Environmental Microbiology 84.

11. Langmead B, Salzberg SL. 2012. Fast gapped-read alignment with Bowtie 2. Nature Methods 9:357-U54.

12. Li H, Handsaker B, Wysoker A, Fennell T, Ruan J, Homer N, Marth G, Abecasis G, Durbin R, Genome Project Data Processing S. 2009. The sequence alignment/map format and SAMtools. Bioinformatics 25:2078-9.

13. Kopylova E, Noe L, Touzet H. 2012. SortMeRNA: fast and accurate filtering of ribosomal RNAs in metatranscriptomic data. Bioinformatics 28:3211-3217.

14. Wood DE, Lu J, Langmead B. 2019. Improved metagenomic analysis with Kraken 2. Genome Biol 20:257.

15. Li D, Liu CM, Luo R, Sadakane K, Lam TW. 2015. MEGAHIT: an ultra-fast single-node solution for large and complex metagenomics assembly via succinct de Bruijn graph. Bioinformatics 31:1674-6.

16. Grabherr MG, Haas BJ, Yassour M, Levin JZ, Thompson DA, Amit I, Adiconis X, Fan L, Raychowdhury R, Zeng Q, Chen Z, Mauceli E, Hacohen N, Gnirke A, Rhind N, di Palma F, Birren BW, Nusbaum C, Lindblad-Toh K, Friedman N, Regev A. 2011. Full-length transcriptome assembly from RNA-Seq data without a reference genome. Nat Biotechnol 29:644-52.

17. Tully BJ, Sachdeva R, Graham ED, Heidelberg JF. 2017. 290 metagenome-assembled genomes from the Mediterranean Sea: a resource for marine microbiology. PeerJ 5:e3558.

18. Martins PD, Frank J, Mitchell H, Markillie LM, Wilkins MJ. 2019. Wetland sediments host diverse microbial taxa capable of cycling alcohols. Appl Environ Microbiol 85:e00189-19.

19. Huang XQ, Madan A. 1999. CAP3: A DNA sequence assembly program. Genome Research 9:868-877.

20. Li W, Godzik A. 2006. Cd-hit: a fast program for clustering and comparing large sets of protein or nucleotide sequences. Bioinformatics 22:1658-9.

21. Sommer DD, Delcher AL, Salzberg SL, Pop M. 2007. Minimus: a fast, lightweight genome assembler. BMC Bioinformatics 8:64.

22. Hyatt D, Chen GL, LoCascio PF, Land ML, Larimer FW, Hauser LJ. 2010. Prodigal: prokaryotic gene recognition and translation initiation site identification. BMC Bioinformatics 11:119.

23. El-Gebali S, Mistry J, Bateman A, Eddy SR, Luciani A, Potter SC, Qureshi M, Richardson LJ, Salazar GA, Smart A, Sonnhammer ELL, Hirsh L, Paladin L, Piovesan D, Tosatto SCE, Finn RD. 2019. The Pfam protein families database in 2019. Nucleic Acids Res 47:D427-D432.

24. Suzek BE, Wang Y, Huang H, McGarvey PB, Wu CH, UniProt C. 2015. UniRef clusters: a comprehensive and scalable alternative for improving sequence similarity searches. Bioinformatics 31:926-32.

25. Buchfink B, Xie C, Huson DH. 2015. Fast and sensitive protein alignment using DIAMOND. Nature Methods 12:59-60.

26. Aramaki T, Blanc-Mathieu R, Endo H, Ohkubo K, Kanehisa M, Goto S, Ogata H. 2020. KofamKOALA: KEGG Ortholog assignment based on profile HMM and adaptive score threshold. Bioinformatics 36:2251-2252.

27. Mayali X, Weber PK, Brodie EL, Mabery S, Hoeprich PD, Pett-Ridge J. 2012. High-throughput isotopic analysis of RNA microarrays to quantify microbial resource use. ISME J 6:1210-21.

28. Mayali X, Weber PK, Mabery S, Pett-Ridge J. 2014. Phylogenetic patterns in the microbial response to resource availability: amino acid incorporation in San Francisco Bay. PLoS One 9:e95842.

29. Mayali X, Weber PK, Nuccio E, Lietard J, Somoza M, Blazewicz SJ, Pett-Ridge J. 2019. Chip-SIP: Stable Isotope Probing analyzed with rRNA-targeted microarrays and NanoSIMS. Methods Mol Biol 2046:71-87.

30. Wilson RM, Hopple AM, Tfaily MM, Sebestyen SD, Schadt CW, Pfeifer-Meister L, Medvedeff C, McFarlane KJ, Kostka JE, Kolton M, Kolka RK, Kluber LA, Keller JK, Guilderson TP, Griffiths NA, Chanton JP, Bridgham SD, Hanson PJ. 2016. Stability of peatland carbon to rising temperatures. Nature Communications 7:13723.

31. Esson KC, Lin XJ, Kumaresan D, Chanton JP, Murrell JC, Kostka JE. 2016. Alpha- and gammaproteobacterial methanotrophs codominate the active methane-oxidizing communities in an acidic boreal peat bog. Applied and Environmental Microbiology 82:2363-2371.

32. Lin X, Tfaily MM, Steinweg JM, Chanton P, Esson K, Yang ZK, Chanton JP, Cooper W, Schadt CW, Kostka JE. 2014. Microbial community stratification linked to utilization of carbohydrates and phosphorus limitation in a boreal peatland at Marcell Experimental Forest, Minnesota, USA. Appl Environ Microbiol 80:3518-30.

33. Lin X, Tfaily MM, Green SJ, Steinweg JM, Chanton P, Imvittaya A, Chanton JP, Cooper W, Schadt C, Kostka JE. 2014. Microbial metabolic potential for carbon degradation and nutrient (nitrogen and phosphorus) acquisition in an ombrotrophic peatland. Appl Environ Microbiol 80:3531-40.

34. Westram R, Bader K, Prüsse E, Kumar Y, Meier H, Glöckner FO, Ludwig W. 2011. ARB: A Software Environment for Sequence Data, p 399-406. *In* Bruijn FJd (ed), Handbook of Molecular Microbial Ecology I doi:<https://doi.org/10.1002/9781118010518.ch46>.

35. Csardi G, Nepusz T. 2006. The igraph software package for complex network research. InterJournal Complex Systems:1695.
